# Structural Determination of Lysosphingomyelin-509 and Discovery of Novel Class Lipids from Patients with Niemann–Pick Disease Type C

**DOI:** 10.3390/ijms20205018

**Published:** 2019-10-10

**Authors:** Masamitsu Maekawa, Isamu Jinnoh, Yotaro Matsumoto, Aya Narita, Ryuichi Mashima, Hidenori Takahashi, Anna Iwahori, Daisuke Saigusa, Kumiko Fujii, Ai Abe, Katsumi Higaki, Shosei Yamauchi, Yuji Ozeki, Kazutaka Shimoda, Yoshihisa Tomioka, Torayuki Okuyama, Yoshikatsu Eto, Kousaku Ohno, Peter T Clayton, Hiroaki Yamaguchi, Nariyasu Mano

**Affiliations:** 1 Department of Pharmaceutical Sciences, Tohoku University Hospital, 1-1 Seiryo-machi, Aoba-ku, Sendai, Miyagi 980-8574, Japan; yotaro.matsumoto.a5@tohoku.ac.jp (Y.M.); saigusa@tohoku.ac.jp (D.S.); yoshihisa.tomioka.a6@tohoku.ac.jp (Y.T.); hiroaki.yamaguchi@med.id.yamagata-u.ac.jp (H.Y.); mano@hosp.tohoku.ac.jp (N.M.); 2 Laboratory of Clinical Pharmacy, Faculty of Pharmaceutical Sciences, Tohoku University, 1-1 Seiryo-machi, Aoba-Ku, Sendai, Miyagi 980-8574, Japan; i.jin.1994.s@gmail.com (I.J.); anna.iwahori.t8@dc.tohoku.ac.jp (A.I.); ai.abe.q2@dc.tohoku.ac.jp (A.A.); 3 Laboratory of Oncology, Pharmacy Practice and Sciences, Graduate School of Pharmaceutical Sciences, Tohoku University, 6-3 Aoba, Aoba-Ku, Sendai, Miyagi 980-8578, Japan; 4 Division of Child Neurology, Tottori University Hospital, 86 Nishi-machi, Yonago, Tottori 683-8503, Japan; aya.luce@nifty.com (A.N.); ohno@sanmedia.or.jp (K.O.); 5 Department of Clinical Laboratory Medicine, National Center for Child Health and Development, 2-10-1 Okura, Setagaya-ku, Tokyo 157-8535, Japan; mashima-r@ncchd.go.jp (R.M.); okuyama-t@ncchd.go.jp (T.O.); 6 Koichi Tanaka Mass Spectrometry Research Laboratory, Shimadzu Corporation, 1 Nishinokyo-Kuwabaracho Nakagyo-ku, Kyoto 604-8511, Japan; th1128ca@shimadzu.co.jp (H.T.); shosei-y@shimadzu.co.jp (S.Y.); 7 Department of Integrative Genomics, Tohoku Medical Megabank Organization, Tohoku University, 2-1 Seiryo-machi, Aoba-ku, Sendai, Miyagi 980-8575, Japan; 8 Department of Psychiatry, Dokkyo Medical University School of Medicine, 880 Kitakobayashi, Mibu, Tochigi 321-0293, Japan; fujii@dokkyomed.ac.jp (K.F.); shimoda@dokkyomed.ac.jp (K.S.); 9 Division of Functional Genomics, Research Centre for Bioscience and Technology, Faculty of Medicine, Tottori University, 86 Nishi-cho, Yonago 683-8503, Japan; kh4060@med.tottori-u.ac.jp; 10 Department of Psychiatry, Shiga University of Medical Science, Setatsukiwacho, Otsu, Shiga 520-2192 Japan; ozeki@belle.shiga-med.ac.jp; 11 Advanced Clinical Research Center, Institute for Neurological Disorders, Furusawa-Miyako 255, Asou-ku, Kawasaki, Kanagawa 215-0026, Japan; yosh@sepia.ocn.ne.jp; 12 Inborn Errors of Metabolism, Clinical and Molecular Genetics Unit, UCL Great Ormond Street Institute of Child Health. 30 Guilford Street, University College London, WC1N 1EH London, UK; peter.clayton@ucl.ac.uk

**Keywords:** Niemann–Pick disease type C, biomarkers, chemical diagnosis, lysosphingomyelin-509, *N*-acyl-phospholipids, LC–MS/MS, HAD–MS/MS, chemical derivatization, identification, structural determination

## Abstract

Niemann–Pick disease type C (NPC) is an autosomal recessive disorder caused by the mutation of cholesterol-transporting proteins. In addition, early treatment is important for good prognosis of this disease because of the progressive neurodegeneration. However, the diagnosis of this disease is difficult due to a variety of clinical spectrum. Lysosphingomyelin-509, which is one of the most useful biomarkers for NPC, was applied for the rapid and easy detection of NPC. The fact that its chemical structure was unknown until recently implicates the unrevealed pathophysiology and molecular mechanisms of NPC. In this study, we aimed to elucidate the structure of lysosphingomyelin-509 by various mass spectrometric techniques. As our identification strategy, we adopted analytical and organic chemistry approaches to the serum of patients with NPC. Chemical derivatization and hydrogen abstraction dissociation–tandem mass spectrometry were used for the determination of function groups and partial structure, respectively. As a result, we revealed the exact structure of lysosphingomyelin-509 as *N*-acylated and *O*-phosphocholine adducted serine. Additionally, we found that a group of metabolites with *N*-acyl groups were increased considerably in the serum/plasma of patients with NPC as compared to that of other groups using targeted lipidomics analysis. Our techniques were useful for the identification of lysosphingomyelin-509.

## 1. Introduction

Niemann–Pick disease type C (NPC) is an autosomal recessive disease characterized by progressive central nervous degeneration [1]. It is considered life-limiting and severe because many patients with NPC become bedridden and develop respiratory failure. Moreover, clinical symptoms of patients with NPC are diverse. A wide range of age-related symptoms and signs are observed, such as systemic symptoms (cholestasis and splenomegaly) in infants and psychiatric presentations in adults as old as 60 years. Misdiagnosis and delayed diagnosis of NPC due to its diversity and rarity could lead to a decrease in the quality of life of patients [2]. In NPC, there is an abnormality in cholesterol traffic due to the functional deficiency of lysosomal membrane protein NPC1 or soluble protein NPC2 involved in cholesterol export [3], resulting in free cholesterol accumulation. Additionally, sphingolipids, such as glycosphingolipids and sphingomyelins, accumulate simultaneously [4]. Although the detailed molecular and pathophysiological mechanisms of NPC are unknown, it is believed that the lipid traffic abnormalities cause NPC pathology, and, therefore, are considered therapeutic targets for NPC. Miglustat, the only marketed drug for NPC, reduces the accumulation of glycosphingolipids by inhibiting glucosylceramide synthase and, as a result, delays and/or suppresses the progression of the central nervous system symptoms [5]. Furthermore, 2-hydroxypropyl-β-cyclodextrin, which eliminates excessive cholesterol, was reported in recent clinical trials to delay the symptoms of the central nervous system in NPC patients [6].

Lipid disruption in this disease is useful in many diagnostic tests. Classically, accumulated cellular cholesterol was detected by filipin staining and was considered a gold standard pathological test for NPC [2]. Gene analysis targeting *NPC1* or *NPC2* through next-generation sequencing is another gold standard used. Recently, oxysterols [7] that are produced by auto-oxidation of cholesterol and the bile acid-like metabolites, probably generated from oxysterols [8,9], were used as biomarkers. Moreover, lysosphingolipids, such as SPC and glucosylsphingosine, were reported as plasma biomarkers [10]. In 2015, lysosphingomyelin-509 (Lyso-SM-509), which was detected at *m/z* 509 and increased significantly in the plasma of patients with NPC, was reported as a useful biomarker for NPC [11]. Although the Lyso-SM-509 peak is widely used for NPC screening and is already included in the clinical practice guideline for NPC [2], its structure was unknown until identification by Sidhu et al. [12]. It suggests the existence of an unknown metabolic pathway in NPC, and its elucidation may lead to the discovery of novel treatment targets. Accordingly, understanding Lyso-SM-509 structure will not only be useful in the development of accurate diagnostic methods using a Lyso-SM-509 standard, but will also reveal a novel aspect of NPC pathophysiology. In this study, we aimed to elucidate the chemical structure of Lyso-SM-509 based on an analytical and organic chemistry approach.

## 2. Result and Discussion

### 2.1. Structural Speculation of Lyso-SM-509

**Figure 1:**
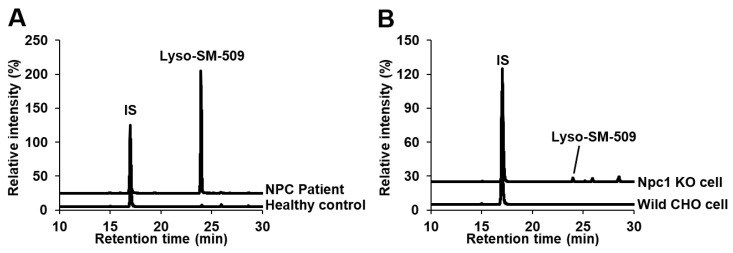
Selected reaction monitoring analysis for searching of Lyso-SM-509. (**A**) Typical total ion current chromatograms of serum. Upper, patient with Niemann–Pick disease type C (NPC); lower, healthy control. (**B**) Typical total ion current chromatograms of cells. Upper, Npc1 (−/−) gene trap CHO cell; lower, CHO cell.

**Figure 2:**
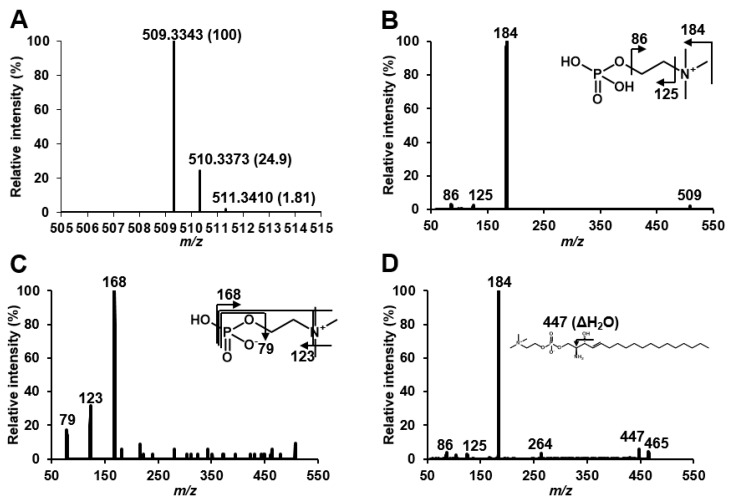
Mass spectra of Lyso-SM-509 and SPC. (**A**) Typical high-resolution mass spectrum of the serum of patient with NPC. (**B**) Typical product ion spectrum of Lyso-SM-509 on positive ion mode. (**C**) Typical product ion spectrum of Lyso-SM-509 on negative ion mode. (**D**) Typical product ion spectrum of SPC on positive ion mode.

**Figure 3:**
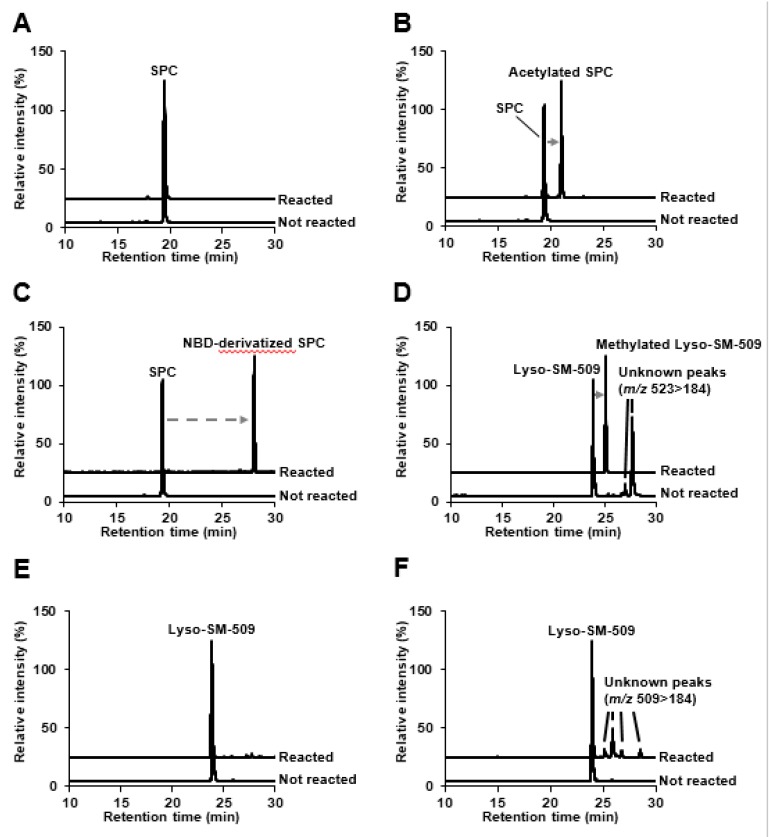
Derivatization for SPC and Lyso-SM-509. (**A**) Selected reaction monitoring (SRM) analysis of methyl esterified SPC. Upper, reacted SPC; lower, SPC. (**B**) SRM analysis of acetylated SPC. Upper, reacted SPC; lower, SPC. (**C**) SRM analysis of NBD-derivatized SPC. Upper, reacted SPC; lower, SPC. (**D**) Estimation of the carboxy group on Lyso-SM-509 by methyl esterification. (**E**) Estimation of the hydroxy group on Lyso-SM-509 by acetylation. (**F**) Estimation of the amine group on Lyso-SM-509 by NBD-derivatization. NBD, 7-nitro-2,1,3-benzoxadiazole; SRM, selected reaction monitoring.

Giese et al. speculated that Lyso-SM-509 might be a carboxylated SPC due to its features that produces ion at *m/z* 184 derived from phosphocholine group, and because the mass difference from SPC is 44 Da, corresponding to carbon dioxide group. We first confirmed the Lyso-SM-509 peak, which was markedly increased in patients with NPC than in healthy controls (Figure 1A), and we aimed to extract it from the NPC model cell. However, the Lyso-SM-509 peak was hardly observed from *Npc1* gene trap CHO cell [13] (Figure 1B). Therefore, we planned to identify it based on an analytical and organic chemistry approach, using the small volume of serum from patients with NPC. The result of high-resolution mass spectrometry agreed with the previous report [11], and the ion formula was determined as C_24_H_50_N_2_O_7_P^+^ (Figure 2A). However, ions characteristics of phosphocholine groups were only observed in low-energy collision induced dissociation in both positive ion mode and negative ion mode (Figure 2B,C). Moreover, we tried to analyze SPC, which generated the dehydrated ion at *m/z* 447, probably derived from the elimination of hydroxy group (Figure 2D). Focusing on the results, we wondered why Lyso-SM-509 did not produce dehydrated ions even the sphingolipids. Accordingly, we hypothesized that Lyso-SM-509 might not be a compound classified as sphingolipid. Therefore, we aimed to investigate its partial structure. Firstly, the functional groups of Lyso-SM-509 were determined using chemical derivatization techniques. We used the reaction of methylation, acetylation, and 7-nitro-2,1,3-benzoxadiazole (NBD) derivatization for confirmation of carboxy group, hydroxy group, and amine group, respectively. Before applying the strategy to Lyso-SM-509, we verified that this approach worked properly by using SPC as a model (Figure 3A–C). As a result, the acetylation and NBD-derivatization proceeded with SPC. The result agreed with the function group of SPC. Subsequently, we tried to determine the function group of Lyso-SM-509. As a result of the derivatizations, the methylation reaction only proceeded in the case of Lyso-SM-509 (Figure 3D–F). This result suggested that Lyso-SM-509 might have a carboxy group and not have a hydroxy and amine group. Accordingly, it is suggested that Lyso-SM-509 might have the different function groups and the skeleton and from SPC.

**Figure 4:**
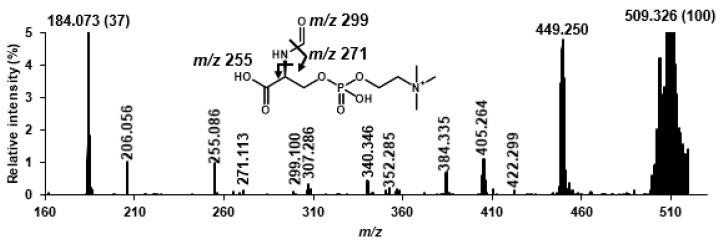
HAD–MS/MS spectrum of Lyso-SM-509. HAD, hydrogen abstraction dissociation; MS/MS, tandem mass spectrometry.

**Figure 5:**
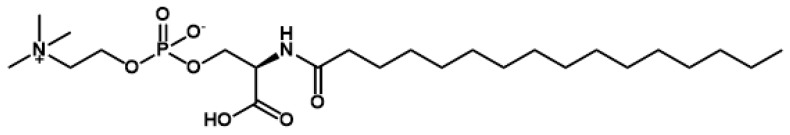
Speculated structure of Lyso-SM-509.

Next, we obtained more structural information using hydrogen abstraction dissociation–tandem mass spectrometry [14], which could obtain richer mass spectrum than the general tandem mass spectrometry. As characteristic ions for speculation of the Lyso-SM-509 structure, *m/z* 255.086, 271.113, and 299.100 were detected in addition to ions by phosphorylcholine group. As a result, it was indicated that Lyso-SM-509 had the partial structures of *N*-acylated and *O*-phosphorylcholine adducted serine (Figure 4). In view of the results of chemical derivatization and HAD–MS/MS (summarized in Figure S1), we speculated that Lyso-SM-509 is a novel class of lipid, *N*-palmitoyl-*O*-phosphocholine-serine, whose unusual skeleton is serine (Figure 5).

### 2.2. Identification of Lyso-SM-509

Next, we synthesized the authentic standard of *N*-palmitoyl-*O*-phosphocholine-serine, and we aimed to identify the unknown metabolite Lyso-SM-509 as a novel lipid based on the principle of metabolite identification by L. W. Summer et al. [15]. The target compound was synthesized according to the scheme shown in Figure S2A. In brief, palmitic acid was condensed with serine benzyl ester hydrochloride (XA), which resulted to intermediate compounds (XB). Further, it yielded to *N*-palmitoyl-*O*-phosphocholine-serine (XE) after reduction of the benzyl ester form (XD) via the phosphorous acid form (XC). All instrument data of all compounds, including the intermediates, could be assigned exactly (Figure S3). Accordingly, the synthesis of *N*-palmitoyl-*O*-phosphocholine-serine succeeded.

**Figure 6:**
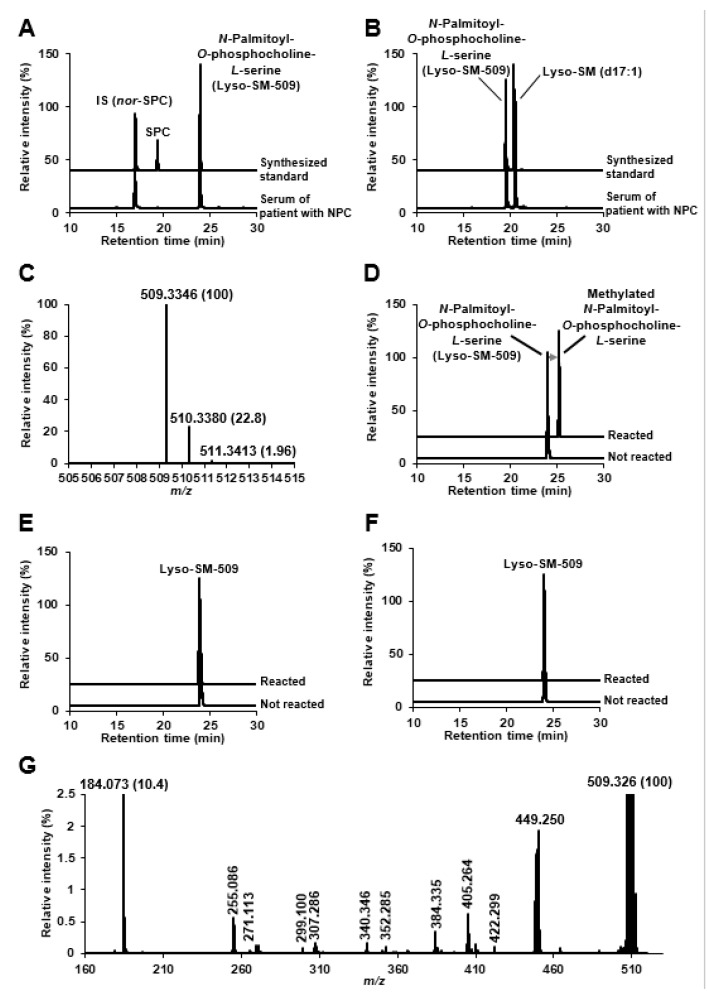
Graphs of Lyso-SM-509. (**A**) Reversed-phase mode chromatographic behavior of synthesized *N*-palmitoyl-*O*-phosphocholine-serine and serum Lyso-SM-509. (**B**) Hydrophilic interaction mode chromatographic behavior of synthesized *N*-palmitoyl-*O*-phosphocholine-serine and serum Lyso-SM-509. (**C**) Typical high-resolution mass spectrum of the synthesized *N*-palmitoyl-*O*-phosphocholine-serine. (**D**) Methyl esterification for *N*-palmitoyl-*O*-phosphocholine-serine. Upper, reacted *N*-palmitoyl-*O*-phosphocholine-serine; lower, *N*-palmitoyl-*O*-phosphocholine-serine. (**E**) Acetylation for *N*-palmitoyl-*O*-phosphocholine-serine. Upper, reacted *N*-palmitoyl-*O*-phosphocholine-serine; lower, *N*-palmitoyl-*O*-phosphocholine-serine. (**F**) NBD-derivatization for *N*-palmitoyl-*O*-phosphocholine-serine. Upper, reacted *N*-palmitoyl-*O*-phosphocholine-serine; lower, *N*-palmitoyl-*O*-phosphocholine-serine. (**G**) Typical hydrogen abstraction dissociation-product ion spectrum of *N*-palmitoyl-*O*-phosphocholine-serine. NBD, 7-nitro-2,1,3-benzoxadiazole.

**Figure 7:**
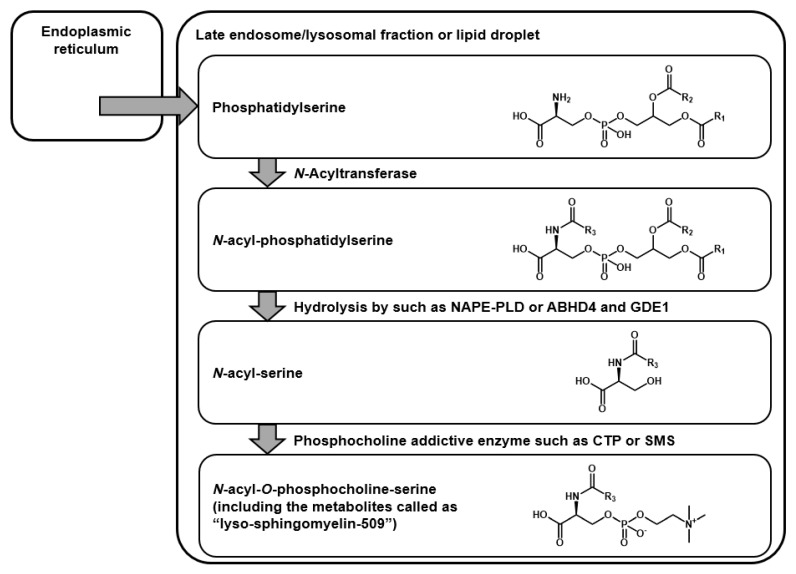
The hypothesis of proposed metabolic pathway of *N*-acyl-*O*-phosphocholine-serines.

Next, the synthesized standard was provided for analyses. In both reversed-phase and hydrophilic interaction chromatography, the chromatographic behavior of the synthesized standard agreed with that of serum Lyso-SM-509 completely (Figure 6A,B). In addition, the accurate mass and the isotopic mass pattern also matched exactly (Figure 6C and Figure 2A). Because the two different orthogonal physical parameters, with accurate mass and retention behaviors of the authentic standard and the unknown substances in clinical specimens, matched completely [15], Lyso-SM-509 was identified as *N*-palmitoyl-*O*-phosphocholine-serine, which is classified as a novel group of lipids. Furthermore, chemical derivatizations for the synthesized standard were repeated in the same way, and all of them agreed with the results of the serum (Figure 6D–F and Figure 3D–F). Likewise, both the product ion spectra on low energy collision-induced dissociation (data not shown) and the spectrum of hydrogen abstraction dissociation–tandem mass spectrometry were equal (Figure 6G and Figure 4). According to the current knowledge of lipid biochemistry, we could not define the biosynthetic pathway of *N*-palmitoyl-*O*-phosphocholine-serine. However, we hypothesized the possible metabolic pathway (Figure 7). Because acidic lysosomal volume is increased in NPC [16], phosphatidylserines might be translocated from endoplasmic reticulum and concentrated as lysosomal fraction, like cholesterol [17]. Subsequently, *N*-acyl-phosphatidylserines are probably metabolized from phosphatidylserine by *N*-acyltransferase [18]. The hydrolysis to *N*-acyl-serines might occur via multiple pathways [19]. A recent report by Van Rooden et al. showed an increase in α/β-hydrolase domain-containing protein, one of the hydrolysing enzymes for *N*-acyl-phosphatidylethanolamine in *Npc1* (-/-) mouse brains [20]. However, there is only one report about the existence of *N*-acyl-phosphatidylserines and *N*-acyl-serines in humans [21]. The addition of phosphocholine to *N*-acyl-serines, including the subcellular location of the metabolism, is also unclear. In the case of glycerophospholipids, phosphatidylcholine is synthesized from diacylglycerol via the Kennedy pathway [22], catalysed by phosphocholine cytidylyltransferase, which is concentrated in the lipid vesicles. In other words, sphingomyelins are biosynthesized from ceramides by sphingomyelin synthase located in the Golgi complex and plasma membrane [23]. Interestingly, the conjugation by phosphocholine was reported for some drugs, such as everolimus [24] and bicycle[1.1.1]pentane [25]; however, the detailed mechanisms were unknown.

### 2.3. Analysis of Plasma/Serum Novel Class Lipids

**Figure 8:**
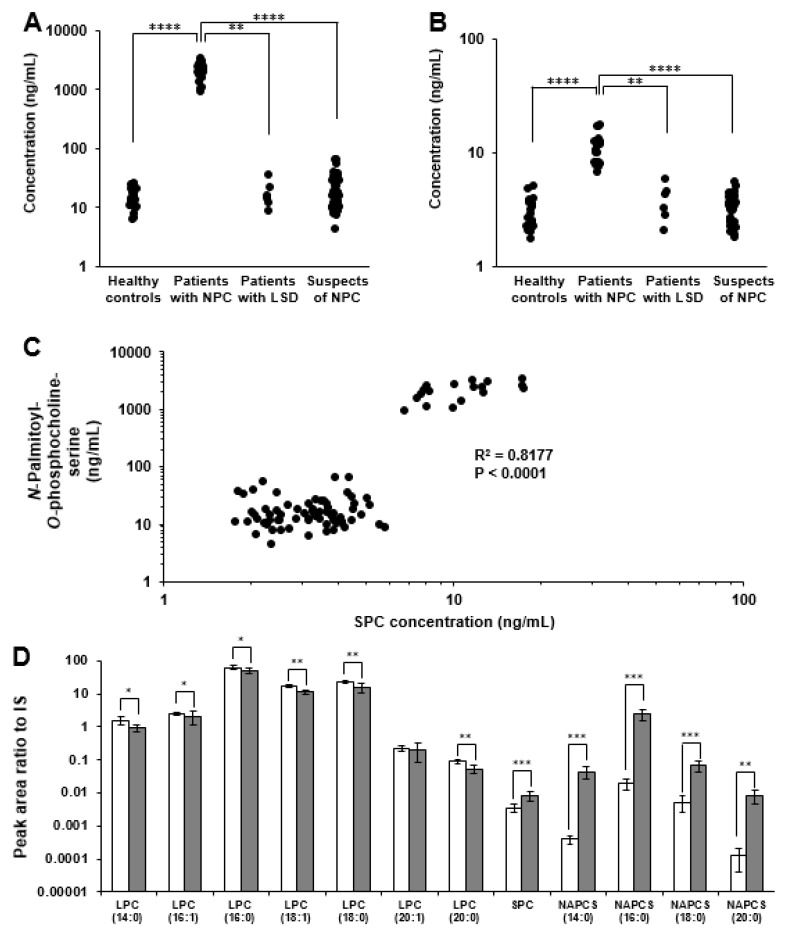
Analyses of various class lipids in serum/plasma. (**A**) The serum/plasma concentration of *N*-palmitoyl-*O*-phosphocholine-serine. ** *p* < 0.01, *** *p* < 0.0001, Steel–Dwass test. (**B**) The serum/plasma concentration of SPC. ** *p* < 0.0001; **** *p* < 0.0001, Steel–Dwass test. (**C**) Correlation of the concentration between SPC and *N*-palmitoyl-*O* phosphocholine-serine. (**D**) Targeted lipidomics for lipids having phosphocholine group. * *p* < 0.05; ** *p* < 0.01; ** *p* < 0.001; Wilcoxon’s test. LSD, lysosomal storage disorder.

Finally, *N*-palmitoyl-*O*-phosphocholine-serine and other related phospholipids in the serum/plasma were analyzed. Partially deuterium substituted *N*-palmitoyl-*O*-phosphocholine-serine was also prepared (the synthetic scheme was shown in Figure S2B) and was used as an internal standard. The concentration of *N*-palmitoyl-*O*-phosphocholine-serine in the serum/plasma of patients with NPC was significantly higher than in all the control groups (Figure 8A). In the patients with other lysosomal storage disorders and those suspected to have NPC but in whom no mutations in *NPC1* or *NPC2* could be detected, the concentrations of *N*-palmitoyl-*O*-phosphocholine-serine were much lower than in patients with NPC (Figure 8A and Table S1). Because no overlap was found in the serum/plasma concentration of *N*-palmitoyl-*O*-phosphocholine-serine between patients with NPC and all controls, both 100% of sensitivity and specificity in receiver operating characteristic analysis was provided (figure not shown). In SPC, the fold change between patients with NPC and control subjects was much smaller than that of *N*-palmitoyl-*O*-phosphocholine-serine (Figure 8B), similar to previous reports [10,11,26,27]; however, the concentrations of SPC and *N*-palmitoyl-*O*-phosphocholine-serine showed good correlation (Figure 8C). The lipid class of SPC and *N*-palmitoyl-*O*-phosphocholine-serine is different; however, their metabolism might correlate with each other via some pathways, indicating that the initial metabolic reaction of sphingolipids is the combination of *L*-serine and palmitoyl-CoA. In addition, we acquired in silico targeted lipidomics analysis the lipids having phosphocholine groups. The result suggested the presence of a group of *N*-acylated-*O*-phosphocholine-serines, and they also increased remarkably similar to the palmitate form (Figure 8D). Moreover, several lysophosphatidylcholine, interestingly, decreased significantly. As a possibility, the consumption or the decrease in lysophosphatidylcholine was suspected. Summarizing our results, it was suggested that novel lipid metabolic disruption in NPC existed. As a possibility, lysosomal fraction may gradually form a lipid droplet [28,29], where lipid and enzymes are concentrated and the abnormal lipid metabolism at the fraction might be stimulated in NPC. Because phosphocholine combined lipids changed drastically as shown in our data, it might affect biological activity such as demyelination by lysophosphatidylcholines [30]. In addition, the lipids with a similar structure, *N*-palmitoyl-serine phosphoric acid, act as the inhibitors of lysophosphatidic acid and affect the cellular calcium homeostasis. *N*-acyl-*O*-phosphocholine-serine might have the inhibitory effect against the usual phospholipids, as well [31]. To elucidate detailed metabolism mechanisms and biological functions of *N*-acyl-*O*-phosphocholine-serine, further studies both in vitro and in vivo are needed. The findings of this present study would certainly provide a new insight on the novel NPC pathology and the new field of lipid metabolisms.

## 3. Materials and Methods

### 3.1. Chemicals and Reagents

Formic acid, free cholesterol enzymatic test kit, trimethylamine, pyridine, sodium sulphate, palmitic acid, and palladium/carbon were purchased from FUJIFILM WAKO Chemicals (Osaka, Japan). Acetone, chloroform, ethanol, filipin complex, 1-hydroxytriazole, Ham′s F-12 medium, iodine, pivaloyl chloride, serine, and toluene were purchased from Nacalai tesque (Kyoto, Japan). Acetonitrile, methanol-d_4_, and chloroform-d were purchased from Kanto Kagaku (Tokyo, Japan). Acetyl chloride, dichloromethane, 1, 3-dicyclohexylcarbodiimide, imidazole, *N*-methylmorpholine, *L*-serine benzyl ester hydrochloride, and phosphorus trichloride were purchased from Tokyo chemical industry Co. (Tokyo, Japan). NBD-F was purchased from DONJINDO laboratories (Kumamoto, Japan). Choline tosylate was purchased from Toronto Research Chemicals (North York, ON, Canada). Foetal bovine serum was purchased from Gibco (Grand island, NY, USA). SPC and Lyso-SM (d17:1) were purchased from Avanti polar lipids, Co. Ltd. (Alabaster, AL, USA). Ultrapure water was prepared with PURELAB ultra (Organo Co. Ltd., Tokyo, Japan) and used in all MS analyses.

### 3.2. Structural Speculation of Lyso-SM-509

Nexera ultra high-performance liquid chromatograph system and InertSustain C18 PEEK column (2.1 mm i.d. × 150 mm, 2 μm, GL sciences, Co. Ltd., Tokyo, Japan) were used for basic chromatographic conditions. A mixture of water and formic acid (100:0.1, *v*/*v*) and a mixture of acetone, acetonitrile, and formic acid (50:50:0.1, *v*/*v*/*v*) were used as mobile phase A and B, respectively. Gradient program was as follows: (B) %, 35 > 35 > 100, time (min), 0 > 8 > 50. Flow rate and column oven temperature were set at 0.4 mL/min and 40 °C, respectively. In the selected reaction monitoring (SRM) analysis, QTRAP6500 hybrid tandem mass spectrometer (SCIEX, Framingham, MA, USA) was used on the positive ion mode. Curtain gas, collision gas, ion spray voltage, turbo gas temperature, nebulizer gas, and turbo gas were set at 20 psi, 12 psi, 5500 V, 700 °C, 50 psi, and 50 psi, respectively. Precursor ions of SRM were shown in Table S2. Product ion of SRM was set at *m/z* 184 for all analyses. Product ion scans ranged from 40 to 600 Da, and collision energy (CE) range was set at 30 V in both polarities. In the HR–MS analysis, we used Orbitrap Fusion (Thermo Fisher Scientific, San Jose, CA, USA). Resolution, RF lens, AGC target, and maximum injection time were set at 500,000, 30%, 2 × 10^5^, and 100 milliseconds, respectively. In the HAD–MS/MS analysis, MALDI-DIT-FP mass spectrometer (Shimadzu Corporation, Kyoto) was used. 2, 5-Dihydroxbenzoic acid (DHB) was used as MALDI matrix, and the spectrum was integrated with 100 shots per spots. For finding Lyso-SM-509 peak from the serum of patients with NPC, SRM analysis was used. Fifty microliters of serum was mixed with 50 μL of 1 μg/mL of Lyso-SM (d17:1) in ethanol/water solution (3:1, *v*/*v*). Fifty microliters of ethanol/water (3:1, *v*/*v*) and 650 μL of acetonitrile were also added to the mixture, and it was centrifuged at 15,000 ×*g* at 4 °C for 10 min. Seven hundred and twenty microliters of supernatant was dried with a centrifugal evaporator at 40 °C. The aliquot was reconstructed with 45 μL of ethanol/water (3:1, *v*/*v*), and 5 μL of the solution was injected into LC/SRM analysis or LC/HR–MS analysis. Lyso-SM (d17:1) was detected at 17.2 min, and the peak speculated as Lyso-SM-509 was much higher in patients with NPC and was detected at 24 min. In the case of cells, Chinese hamster ovary (CHO) cells and *Npc1* (−/−) gene trap CHO cells [13] were cultured in Ham′s F-12 containing 10% of foetal bovine serum. Cholesterol accumulation in NPC model cells was verified through filipin staining [32], while the free cholesterol determination was by enzymatic assay kit (data not shown). Fifty microliters of cell suspension (10^7^ cells/mL suspended with PBS) or cell-cultured medium was treated and analyzed similarly as in the case of serum mentioned above. In HR–MS analysis, theoretical mass of lysosphingomyelin-509 (formula: C_24_H_50_N_2_O_7_P^+^) and the isotopic mass are 509.3350, 510.3384, and 540.3420, respectively. In HAD–MS/MS analysis, the serum of a patient with NPC was fractionated with Bond Elut C_18_ cartridge (100 mg, 1 cc, Agilent technologies, Santa Clara, CA). Two hundred microliter of serum was diluted with water was loaded onto the cartridge. After washing with 1 mL of water and 1 mL of methanol/water (1:3, *v*/*v*), the fraction was taken with 1 mL of methanol. Once the liquid content has been evaporated by centrifugal evaporator, the aliquot was reconstructed with ethanol/water (1:1, *v*/*v*) and mixed with a matrix. The mixture was spotted onto the MALDI-plate and analyzed as the conditions mentioned above.

### 3.3. Chemical Derivatization for Lyso-SM-509 and SPC

The serum from patients with NPC was pre-treated for protein precipitation and the dried aliquot was provided for chemical derivatization. The treated samples were provided for LC/SRM analysis and all product ions were set at *m/z* 184. Methyl esterification reaction of carboxy group was acquired using methanol-hydrochloride [33]. One hundred microliters of acetyl chloride was mixed with 5 mL of methanol and the mixture served as HCl-methanol solution. Fifty microliters of 1 μg/mL of standard solution or protein precipitated serum aliquot was once evaporated and was added 100 μL of HCl-methanol solution. The mixture was stood at 80 °C for 60 min and dried up under nitrogen gas stream. The dried mixture was reconstructed with 50 μL of methanol, and 1 μL of the aliquot was analyzed by LC/SRM. For SRM of SPC and the reacted SPC (Figure 2A), precursor ions were set at *m/z* 465.3 (SPC) and *m/z* 479.3 (If methyl esterified). In the case of Lyso-SM-509 (Figure 2D and Figure 3D), precursor ions were set at *m/z* 509.3 (Lyso-SM-509) and *m/z* 523.3 (If methyl esterified). For acetyl esterification to the hydroxy group, acetyl chloride reagent was used [34]. One microgram per milliliter of standard solution or protein-precipitated serum was once evaporated. Fifty microliters of acetonitrile, 25 μL of acetic acid, and 25 μL of acetyl chloride were added to the sample and stood at 40 °C for 20 min. The reaction mixture was neutralized with 5% of sodium hydrogen carbonate and then evaporated. Fifty microliters of methanol was added and analyzed. For SPC (Figure 2B), precursor ions were set at *m/z* 465.3 (SPC) and *m/z* 507.3 (if acetylated), while for Lyso-SM-509 (Figure 2E and Figure 3E), precursor ions for monitoring were set at *m/z* 509.3 (Lyso-SM-509) and *m/z* 551.3 (if acetylated). For amine group, NBD-derivatization was used [35,36]. In brief, 50 μL of sample solution was once dried up and reconstructed with 50 μL of 400 mmol/L of sodium borate buffer (pH 8.0). Fifty microliters of 40 mmol/L of NBD-F in acetonitrile was added and stood at 60 °C for 2 min. After evaporation, the reaction mixture was constructed with 50 μL of methanol/water (1:1, *v*/*v*), and 1 µL of aliquot was analyzed. For SPC (Figure 2C), precursor ions were set at *m/z* 465.3 (SPC) and *m/z* 628.3 (if NBD-derivatized) while for Lyso-SM-509 (Figure 2F and Figure 3F), precursor ions were set at *m/z* 509.3 (Lyso-SM-509) and *m/z* 672.3 (if NBD-derivatized).

### 3.4. Chemical Synthesis of N-Palmitoyl-O-Phosphocholine-Serine

All NMR spectra were recorded with a JEOL ECA-600 (600 MHz) spectrometer (Tokyo, Japan) and dissolved in CDCl_3_ or CD_3_OD. Chemical shifts and coupling constants were expressed in δ (ppm) and hertz (Hz), respectively. NMR spectra were referenced as tetramethylsilane (0 ppm) in CDCl_3_ or as MeOH (3.31 ppm) in CD_3_OD as an internal standard. The following abbreviations were used: s = singlet, d = doublet, t = triplet, quint = quintet, m = multiplet, and brs = broad singlet. ^1^H- and ^13^C-NMR chemical shift assignments were made based on ^1^H-^1^H COSY, ^1^H-^13^C HMQC, and ^1^H-^13^C HMBC experiments and shown in Figure S3. HR–MS was obtained from Orbitrap Fusion mass spectrometer. The chemical synthetic scheme was a designed reference of some literatures and is shown in Figure S3.

(*S*)-*N*-palmitoyl-serine benzyl ester (XB): The procedures reported by Kim, et al. [37] were modified as described below. A solution of palmitic acid (1.80 g, 7.0 mmol) in dichloromethane (64 mL) at 0 °C was added to a mixture of *L*-serine benzyl ester hydrochloride (XA, 1.5 g, 6.4 mmol), 1-hydroxybenzotriazole (0.95 g, 7.05 mmol), and 1,3-dicyclohexylcarbodiimide (1.46 g, 7.16 mmol). *N*-methylmorpholine was added to this mixture (1.5 mL, 13.5 mmol). The mixture was stirred at 0 °C for 1 hour, followed by stirring at 25 °C overnight. The insoluble material was filtered, and the filtrate was concentrated. Chromatography on silica gel (dichloromethane/acetone, 100/0 to 85/15, v/v) gave 1.86 g (67%) of the product as a white solid. HR–MS calculated for [C_26_H_43_NO_4_+H]^+^ ([M+H]^+^) 434.3265, gave 434.3263.

(*S*)-*N*-palmitoyl-*O*-phosphonate-serine benzyl ester (XC): The procedures reported by Bartel et al. [38] were modified as described below. To a solution of imidazole (1.88 g, 26.55 mmol) in 40 mL of dry acetonitrile, phosphorous trichloride (1.045 g; 0.67 ml; 7.61 mmol) followed by triethylamine (2.62 g; 3.6 mL; 25.85 mmol) were added in drops under argon atmosphere. After stirring for 15 min, a solution of XB (0.76 g, 1.77 mmol) in 100 mL toluene (warm up to 60 °C and return to room temperature to dissolve if necessary) was added in drops to the mixture over a period of 25 min and then left overnight at room temperature. The reaction stopped by the addition of 20 mL water, and the mixture was stirred further for 30 min. The solvent was evaporated in a vacuum, and then 100 mL of pyridine/triethylamine (80:20, v/v) was added, and the solvent evaporated again. The crude product was dissolved in 100 mL of chloroform and washed with 50 mL of water and 50 mL of brine. The organic layer was separated, and the aqueous layer was washed with 300 mL of chloroform. After drying, the collected organic layer with sodium sulphate and a white waxy product of the evaporated solvent in a vacuum were obtained without further purification. HR–MS calculated for [C_26_H_44_NO_6_P–H]^–^ ([M–H]^–^) 496.2833 and gave 498.2827.

(*S*)-*N*-palmitoyl-*O*-phosphocholine-serine benzyl ester (XD): The procedures of Bartel et al. [37] were modified as described below. *N*-palmitoyl serine phosphonate benzyl ester (XC, 1.77 mmol) and choline tosylate (0.98 g; 3.54 mmol) were dissolved in 20 mL of dry pyridine, and pivaloyl chloride (0.43 mL; 3.54 mmol) was added. Iodine (0.90 g; 3.54 mmol) dissolved in 20 mL of pyridine/water (98:2, *v*/*v*) was added for over 15 min to the mixture being stirred, and stirring continued for 5 min. The solution was mixed with 80 mL of chloroform, 70 mL of 5% aqueous sodium hydrogen sulphate, and 50 mL of methanol. After shaking in a separation funnel, the organic layer was separated, and the aqueous layer was washed with 330 mL of chloroform. The collected organic layers were dried with sodium sulphate, and the solvent was evaporated in a vacuum. Traces of pyridine and water were removed by repeated evaporation, and yellowish oil was yielded with dry toluene. The crude product was purified by chromatography on silica gel (dichloromethane/methanol/water 35:15:0 to 35:15:5, *v*/*v*/*v*). The product was 373 mg (35.1%) as a white solid. HR–MS calculated for [C_31_H_55_N_2_O_7_P+H]^+^ ([M+H]^+^) 599.3820, gave 599.3819.

(*S*)-*N*-palmitoyl-*O*-phosphocholine-serine (XE, called as Lyso-SM-509): A solution of *N*-palmitoyl-*O*-phosphocholine-serine benzyl ester (XD, 51.6 mg, 86 μmol) in methanol (2 mL) was added to 10% Pd/C (6.1 mg, 12 μmol). The reaction mixture was stirred under hydrogen atmosphere (1 atm) at room temperature for 3 hours. The insoluble material was filtered, and the filtrate was concentrated. Bond-Elut C_18_ cartridge (600 mg, 6 cc) was equilibrated with methanol and water (10 mL each), and then the mixture dissolved in 10% methanol was added. After washing with 10 mL of water, the fraction was eluted with 10 mL of methanol. The solvent was distilled off to obtain colorless powder (40 mg, 91.3%). HR–MS calculated for [C_24_H_49_N_2_O_7_P+H]^+^ ([M+H]^+^) 509.3350, and gave 509.3348.

Choline-d_3_ tosylate: A previous report [37] was modified slightly. To a solution of P-toluenesulfonyl chloride (also called as tosyl chloride; 1.5 g, 8.0 mmol) in THF (5.8 mL), methanol-d_4_ (580 mg, 16 mmol) and 20% sodium hydroxide aqueous solution (4.0 mL) were added at 0 °C. After 4 hours, the mixture was diluted with 20 mL of water and extracted with 20 mL of diethyl ether three times. The combined organic phase was subsequently washed with saturated aqueous ammonium chloride and brine. The organic layer was dried over anhydrous sodium sulphate and concentrated in a vacuum. After substitution to argon atmosphere in the reaction-pod, 0.75 mL of *N*,*N*-dimethyl ethanolamine and 50 mL of THF were added to the mixture and stirred. The methyl-d_3_-tosylate (1.42 g) in 12.5 mL of THF was added in drops and stirred at room temperature overnight. After evaporation of the solvent and recrystallization in acetonitrile, choline-d_3_ tosylate crystal was obtained (1.42 g).

(*S*)-*N*-palmitoyl-*O*-phosphocholine-serine-d_3_ benzyl ester (XF): *N*-palmitoyl-*O*-phosphonate-serine benzyl ester (XC, 1.77 mmol) and choline-d_3_ tosylate (0.98 g; 3.54 mmol) were dissolved in 20 mL of dry pyridine, and pivaloyl chloride (0.43 ml; 3.54 mmol) was added. The following procedure was the same as the synthesis of (*S*)-*N*-palmitoyl-*O*-phosphocholine-serine benzyl ester. Finally, 373 mg (35.1%) of the product was obtained as a white solid. HR–MS calculated for [C_31_D_3_H_52_N_2_O_7_P+H]^+^ ([M+H]^+^) 602.4008, and gave 602.4005.

(*S*)-*N*-palmitoyl-*O*-phosphocholineserine-d_3_ (XG): A solution of *N*-palmitoyl-*O*-phosphocholine-serine benzyl ester-d_3_ (XF, 51.6 mg, 86 μmol) in methanol (2 mL) was added to 10% Pd/C (6.1 mg, 12 μmol). The following procedure was the same as the synthesis of *N*-palmitoyl-*O*-phosphocholine-serine (XE). Finally, colorless powder was obtained (40 mg, 91.3%). HR–MS calculated for [C_24_D_3_H_46_N_2_O_7_P+H]^+^ ([M+H]^+^) 512.3539, and gave 512.3536.

### 3.5. Identification of Lyso-SM-509

The synthesized *N*-palmitoyl-*O*-phosphocholine-serine (XE) was dissolved in ethanol/water (3:1, *v*/*v*) at 100 μg/mL concentration as stock solution. Stock solution was diluted to 1 μg/mL with ethanol/water (3:1, *v*/*v*). Fifty microliters of the standard solution or the serum of patients with NPC was mixed with the same volume of 1 μg/mL of Lyso-SM (d17:1) and ethanol/water (3:1, *v*/*v*). The pre-treatment procedure for protein precipitation was the same as the procedure described above in the section for the “Structural Speculation of Lyso-SM-509.” One microliter of aliquot sample was provided for analysis. MS/MS conditions, such as the parameters for the ion source probe, were the same as that seen in the “Structural Speculation of Lyso-SM-509 and Chemical Derivatization for Lyso-SM-509 and SPC” section. In SRM analysis, precursor ions were set at *m/z* 465.3 (for SPC), *m/z* 451.3 (for LysoSM (d17:1)), and *m/z* 509.3 (for *N*-palmitoyl-*O*-phosphocholine-serine, also called as Lyso-SM-509). Flow rate and column oven temperature were set at 0.4 mL/min and 40 °C, respectively. In the reversed-phase chromatographic condition, InertSustain PEEK C18 column (2.1 mm i.d. × 150 mm, 2 μm) was used. Formic acid/water (100:0.1, *v*/*v*) and formic acid/acetone/acetonitrile (0.1:50:50, *v*/*v*/*v*) were used as mobile phase A and B, respectively. Time program was set as follows: B (%), 35 > 68 > 68, time (min), 0 > 8> 30. In hydrophilic interaction chromatography mode, YMC-Triart Diol-HILIC column (2.1 mm i.d. × 150 mm, 3 μm, YMC Co., Ltd., Kyoto, Japan) was used. Formic acid/water (100:0.1, *v*/*v*) and formic acid/acetonitrile (0.1:100, v/v) were used as mobile phase A and B, respectively. Gradient program was as follows: B (%), 95 > 65, time (min), 0 > 30. All MS conditions and chemical-derivatization procedures were the same as described above.

### 3.6. Simultaneous Analysis of N-Palmitoyl-O-Phosphocholine-Serine and SPC in the Serum or Plasma

API 5000 tandem mass spectrometer (SCIEX) and Nexera ultra high-performance liquid chromatograph system were used. InertSustain C18 PEEK column (2.1 mm i.d. × 150 mm) was used as an analytical column. Formic acid/water (0.1:100, *v*/*v*) and formic acid/acetonitrile/acetone (0.1:50:50, *v*/*v*/*v*) were used as mobile phase A and B, respectively, at 0.4 mL/min flow rate. Gradient program is as follows: B (%), 25 > 70, time (min), 0 > 30. Curtain gas, collision gas, ion spray voltage, turbo gas temperature, nebulizer gas, and turbo gas were set at 20 psi, 12 psi, 4500 V, 700 °C, 40 psi, and 70 psi, respectively. Detailed SRM transitions were shown in Table S2. Assay validation was done according to the FDA guidance [39]. SCIEX OS-Q software (SCIEX) was used for data analysis. All validation data were summarized in Table S3, and all results were shown in Table S1. Healthy controls, *n* = 20; patients with NPC, *n* = 15; patients with LSD, *n* = 6; and NPC suspected patients, *n* = 44. The age of the different groups was not significantly different; however, there were significantly more male subjects in healthy controls than in the other groups. The significant change in the blood concentrations of *N*-palmitoyl-*O*-phosphocholine-serine or SPC was investigated using Steel–Dwass test, which is used for non-parametric tests among multiple groups. For receiver operating characteristic analysis, a logistic regression model was used for both compounds. Correlation of the concentrations of *N*-palmitoyl-*O*-phosphocholine-serine and SPC was investigated with a single regression analysis model.

### 3.7. Targeted Lipidomics Analysis

Targeted lipidomics analysis was performed using QTRAP6500 mass spectrometer combined with Nexera UHPLC system. General LC, MS/MS, and sample preparation conditions were the same as those in the section of “identification of Lyso-SM-509”. SRM conditions, retention times, and IS for targeted peaks are shown in Table S4. Healthy controls, *n* = 7, Patients with NPC, *n* = 10. There was a similarity between their age and gender. The statistical analysis of peak area ratio was investigated with Wilcoxon’s t-test.

## Supplementary Materials

Supplementary materials can be found at https://www.mdpi.com/1422-0067/20/20/5018/s1.

## Abbreviations

CHO	Chinese hamster ovary
Lyso-SM	Lysosphingomyelin
Lyso-SM-509	Lysosphingomyelin-509
NBD	7-Nitro-2,1,3-benzoxadiazole
NPC	Niemann–Pick disease type C
SPC	Sphingosylphosphocholine
SRM	Selected reaction monitoring
AcOEt	Ethyl acetate
CDCl3	Chloroform-d
CD3OD	Methanol-d_4_
DCC	*N, N*’-Dicyclohexylcarbodiimide
DHB	2, 5-Dihydroxbenzoic acid
Et3N	Triethylamin
HOBt	1-Hydroxybenzotriazole
MeOH	Methanol
NMM	*N*-methylmorphorine
Ph	Phenyl
THF	Tetrahydrofuran
