# Supplementary material for: Structural Determination of Lysosphingomyelin-509 and Discovery of Novel Class Lipids from Patients with Niemann–Pick Disease Type C
Source: Int J Mol Sci. 2019 Oct 10;20(20):5018. doi: 10.3390/ijms20205018 (PMC6829288; doi:10.3390/ijms20205018)
Supplement: Supplementary file 1 [file ijms-20-05018-s001.pdf]

M. Maekawa, I. Jinnoh, Y. Matsumoto, *et al.*, Fig. S1

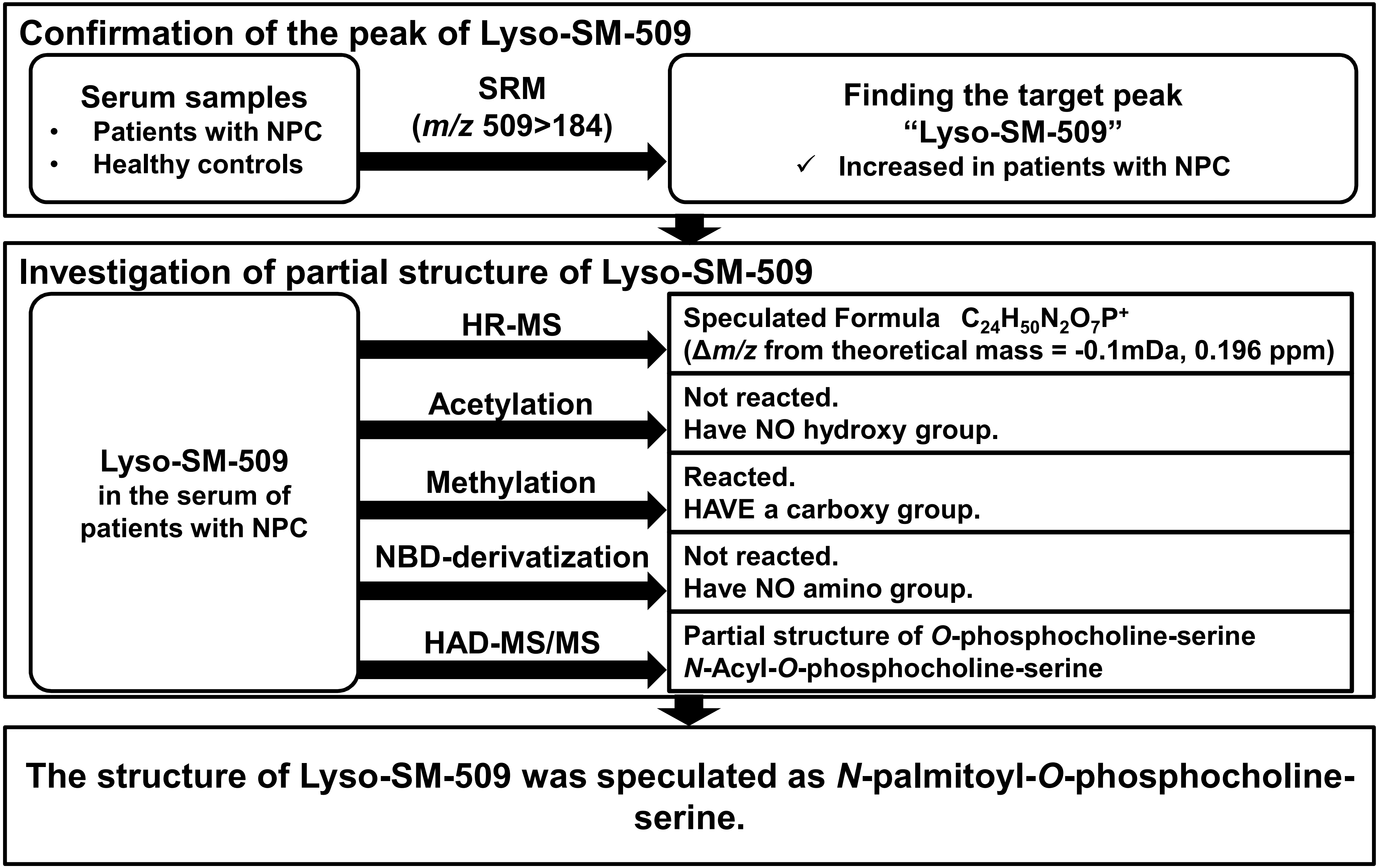

Abbreviations: HAD, hydrogen abstraction dissociation; HR-MS high resolution mass spectrometry; MS/MS, tandem mass spectrometry; NBD, 7-nitro-2,1,3-benzoxadiazole; Lyso-SM-509, lyso-sphingomyelin-509; NPC, Niemann-Pick disease type C; 4-fluoro-7-nitro-2,1,3-benzoxadiazole; SRM, selected reaction monitoring.

A

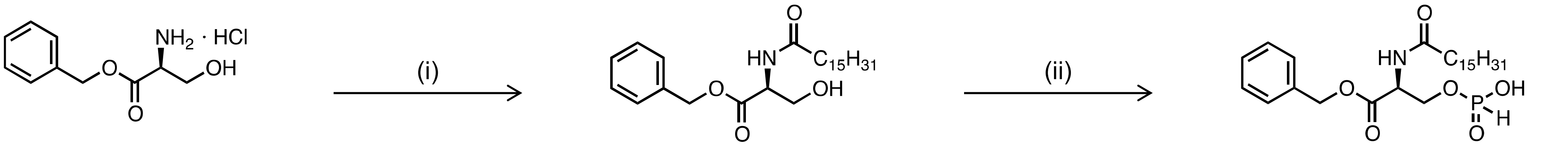

L-serine benzyl ester hydrochloride (**XA**)

(*S*)-*N*-palmitoyl-serine benzyl ester (**XB**)

(*S*)-*N*-palmitoyl-*O*-phosphonate-serine benzyl ester (**XC**)

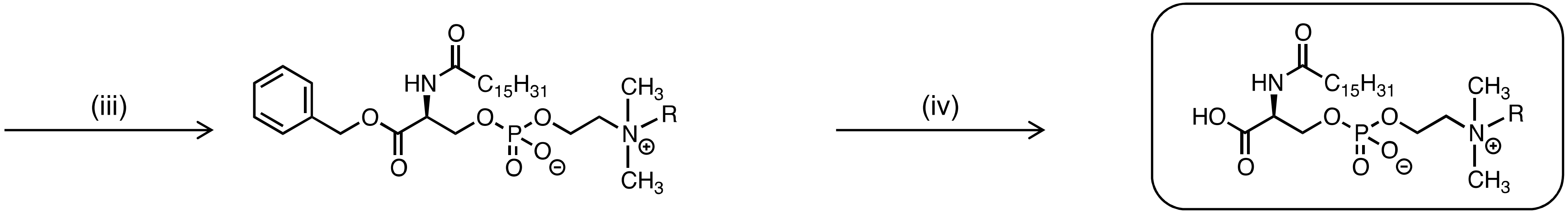

R = CH_3: (*S*)-*N*-palmitoyl-*O*-phosphocholine-serine benzyl ester (**XD**)
R = CD_3: (*S*)-*N*-palmitoyl-*O*-phosphocholine-serine benzyl ester-d_3 (**XF**)

R = CH_3: (*S*)-*N*-palmitoyl-*O*-phosphocholine-serine (Lyso-SM-509) (**XE**)
R = CD_3: (*S*)-*N*-palmitoyl-*O*-phosphocholine-serine-d_3 (**XG**)

Synthesis of (*S*)-*N*-palmitoyl-*O*-phosphocholine-serine and (*S*)-*N*-palmitoyl-*O*-phosphocholine-serine-d_3.
Reagents and conditions: (i) palmitic acid, DCC, HOBt, NMM, CH_2Cl_2, rt, 4 hr; (ii) PCl_3, Imidazole, Et_3N, CH_3CN, toluene, rt, 7 hr; (iii) (a) choline tosylate or choline tosylate-d_3, pyridine, rt, 15 min, (b) I_2, pyridine, H_2O, rt, 5 min; (iv) Pd/C, H_2, AcOEt, rt, 6 hr

B

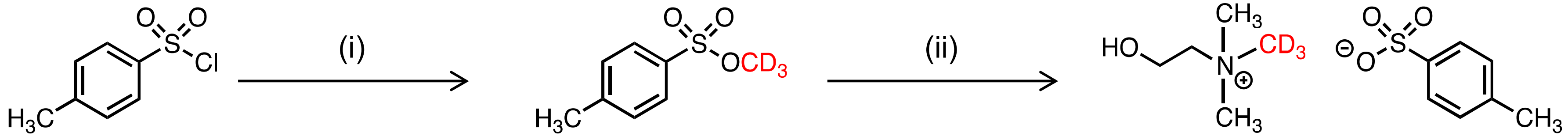

Tosyl chloride

Methyl tosylate-d_3

Choline tosylate-d_3

Synthesis of choline tosylate-d_3.
Reagents and conditions: (i) CD_3OD, NaOH, H_2O, THF, rt, 10 hr; (ii) N,N-dimethylaminoethanol, THF, rt, 24 hr

M. Maekawa, I. Jinnoh, Y. Matsumoto, et al., Fig. S3

A

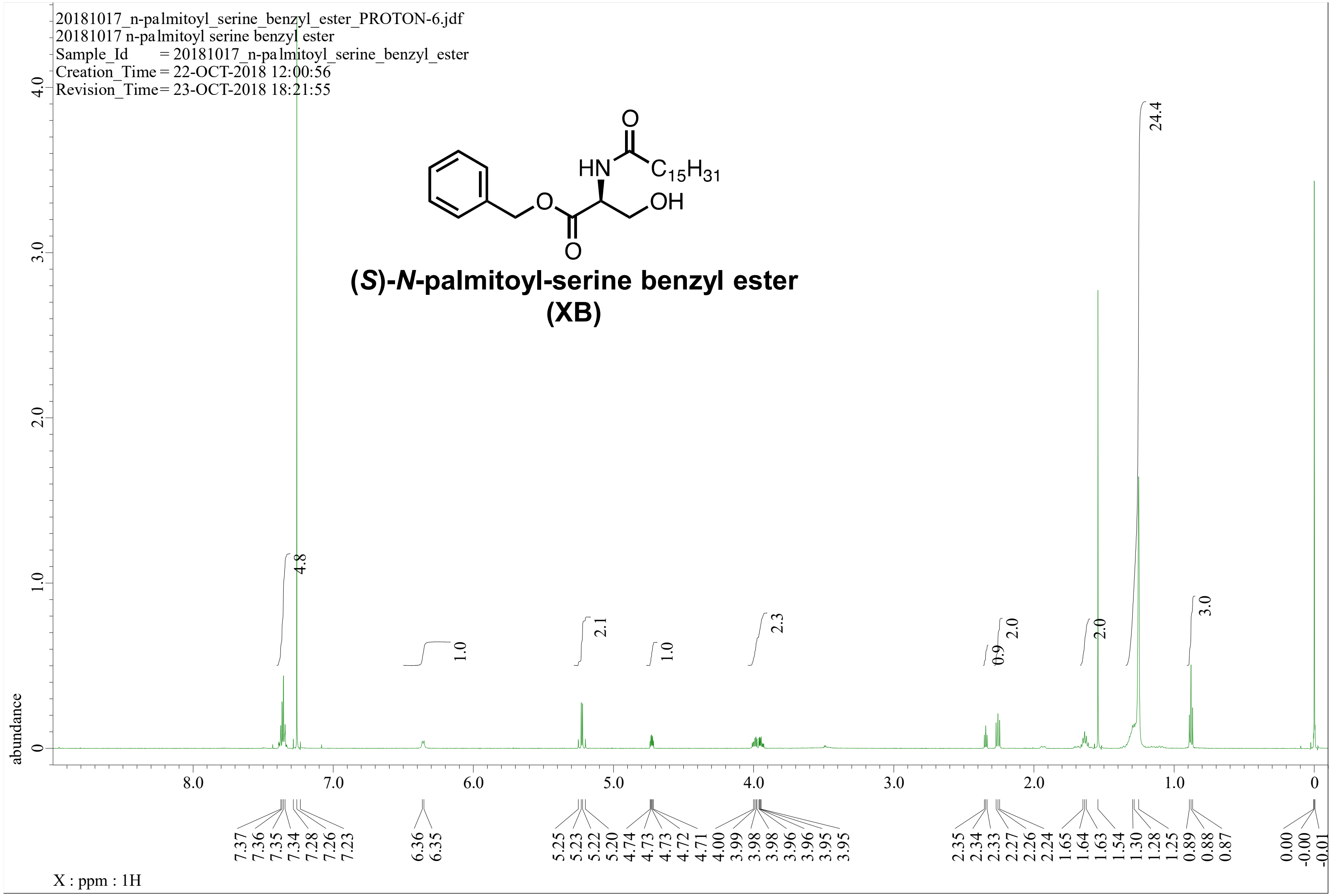

Fig. S3NMR spectrum of synthesized compounds.

a, ^{1}H-NMR spectrum of (*S*)-*N*-palmitoyl-serine benzyl ester (XB) in $CDCl_3$

δ : 0.87–0.89 (t, 3H, J = 6.9 Hz, $-CH_3$), 1.25–1.30 (m, 24H, palmitoyl methylene proton), 1.63–1.65 (m, 2H, NHCO-CH_2-CH_2), 2.24–2.27 (t, 2H, J = 7.6 Hz, NHCO-CH_2), 2.33–2.35 (t, 1H, J = 5.8 Hz, -OH), 3.93–4.01 (m, 2H, COC(N)H-CH_2OH), 4.71–4.74 (m (quint), 1H, J = 3.4 Hz, CO-CH-N), 5.20–5.25 (dd, 2H, J = 17.9, 12.4 Hz, PhCH_2O-CO-), 6.35–6.36 (d, 1H, J = 6.9 Hz, C-NH-CO-), 7.23–7.37 (m, 5H, Ph).

B

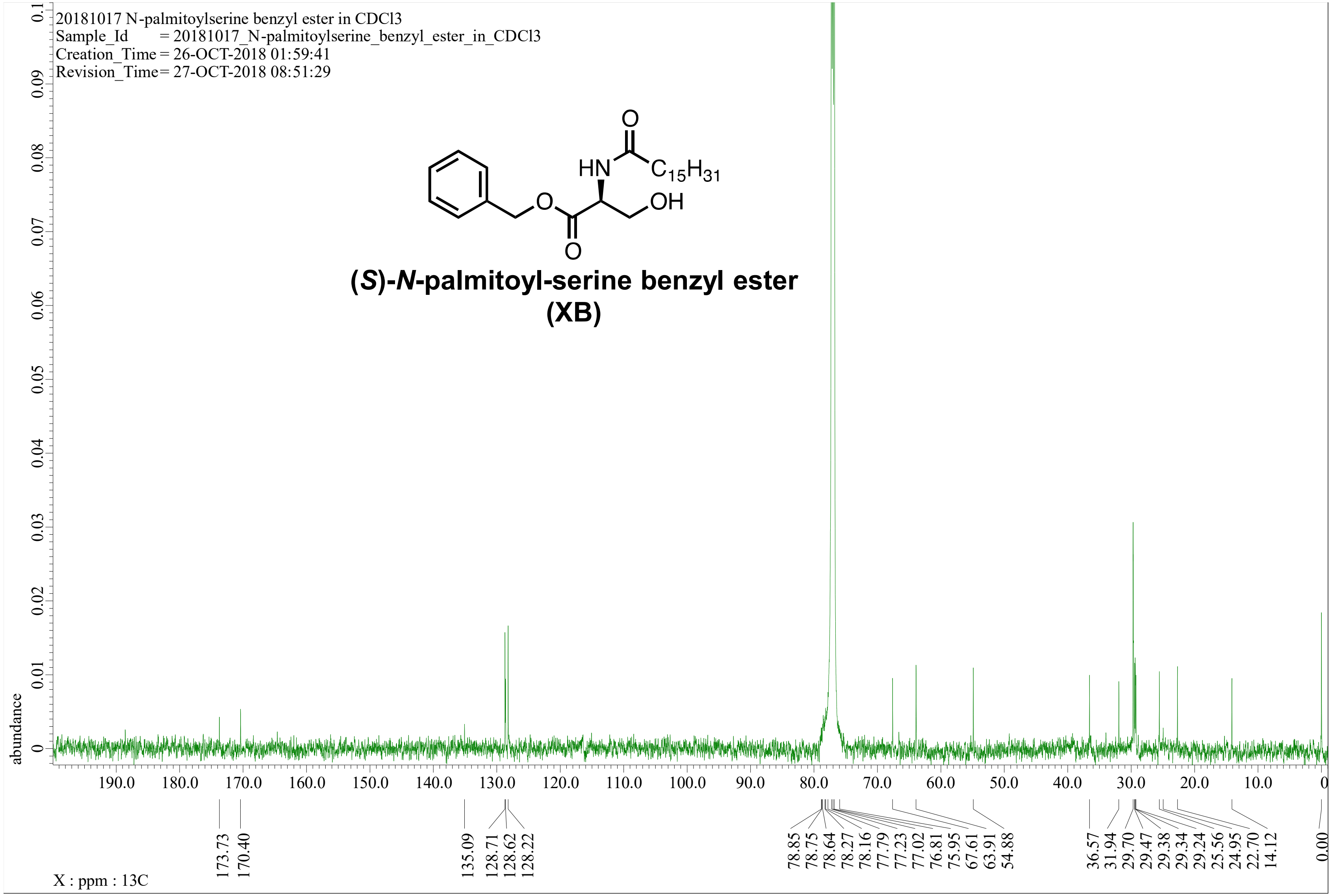

Fig. S3(continued).

b, ^{13}C-NMR spectrum of (*S*)-*N*-palmitoyl-serine benzyl ester (XB) in $CDCl_3$.

δ: 14.1 ($-CH_3$), 22.7 ($-CH_2CH_3$), 25.6 ($-NHCOCH_2CH_2-$), 29.2–29.7 (multiple peaks in the range), 31.9 ($-CH_2CH_2CH_3$), 36.6 ($-NHCOCH_2-$), 54.9 (-NHCH-), 63.9 (-NHCH(-COO)CH_2OH), 67.6 (-Bn), 128.2 (Ph), 128.6 (Ph), 128.7 (Ph), 135.1 (quaternary carbon in Ph), 170.4 (BnOCO-), 173.7 (-NHCO-).

C

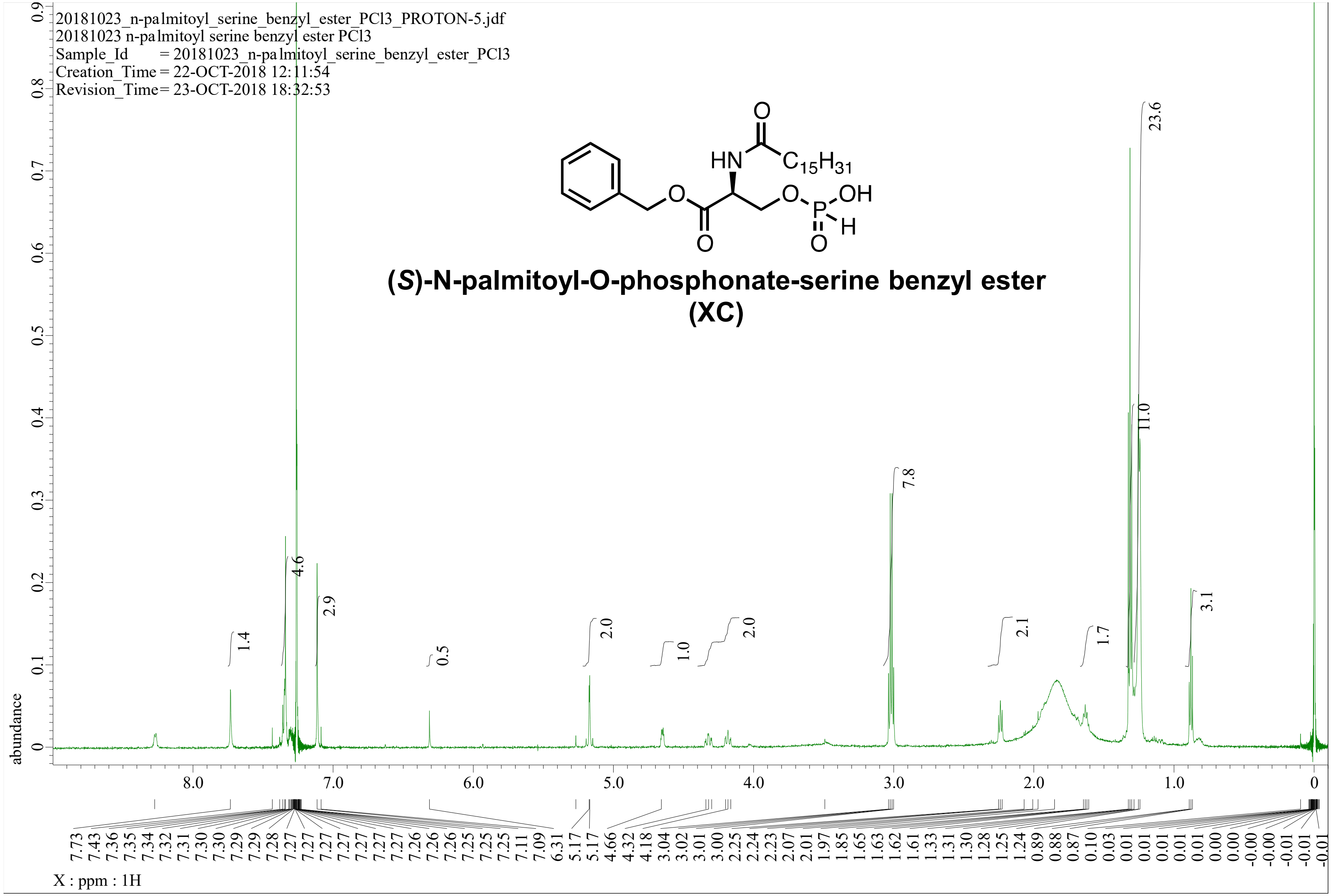

Fig. S3 (continued)

c, ^{1}H-NMR spectrum of (*S*)-*N*-palmitoyl-*O*-phosphonate-serine benzyl ester (XC) in $CDCl_3$.

δ: 0.87–0.89 (t, 3H, J= 6.9 Hz, -CH_3), 1.24–1.33 (m, 24H, palmitoyl), 1.61–1.66 (m, 2H, NCO-CH_2-CH_2), 2.23–2.25 (t, 2H, J= 7.6 Hz, NCO-CH_2), 4.16–4.35 (m, 2H, COCH (-NH) CH_2OH), 4.66 (s, 1H, COCHN), 5.17 (dd, 2H, J= 12.7, 15.5 Hz, Bn-CH_2-OCO), 6.31 (s, 1H, NH), 7.34–7.36 (m, 5H, Bn).

D

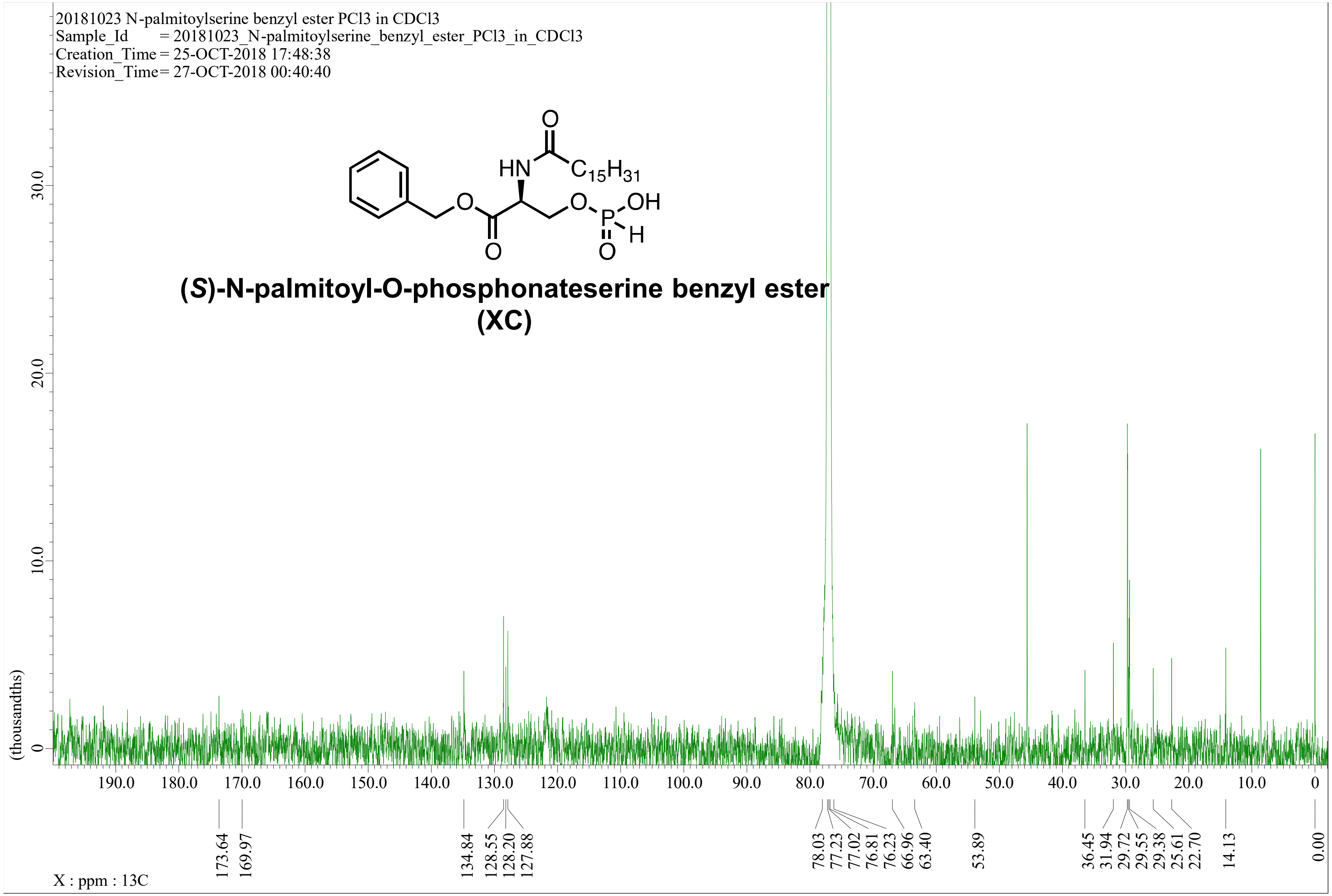

Fig. S3(continued)

d, ^{13}C-NMR spectrum of (*S*)-*N*-palmitoyl-*O*-phosphonateserine benzyl ester (XC) in $CDCl_3$.

δ: 14.1 (-CH_3), 22.7 (-CH_2CH_3), 25.6 (-$NHCOCH_2CH_2$-), 29.4–29.7 (multiple peaks in the range), 31.9 (-$CH_2CH_2CH_3$), 36.5 (-$NHCOCH_2$-), 53.9 (-NHCH-), 63.4 (-NHCH(-COO)CH_2OH), 67.0 (BnCH_2-), 127.9 (Ph), 128.2 (Ph), 128.6 (Ph), 134.8 (quaternary carbon in Ph), 170.0 (BnOCO-), 173.6 (-NHCO-).

E

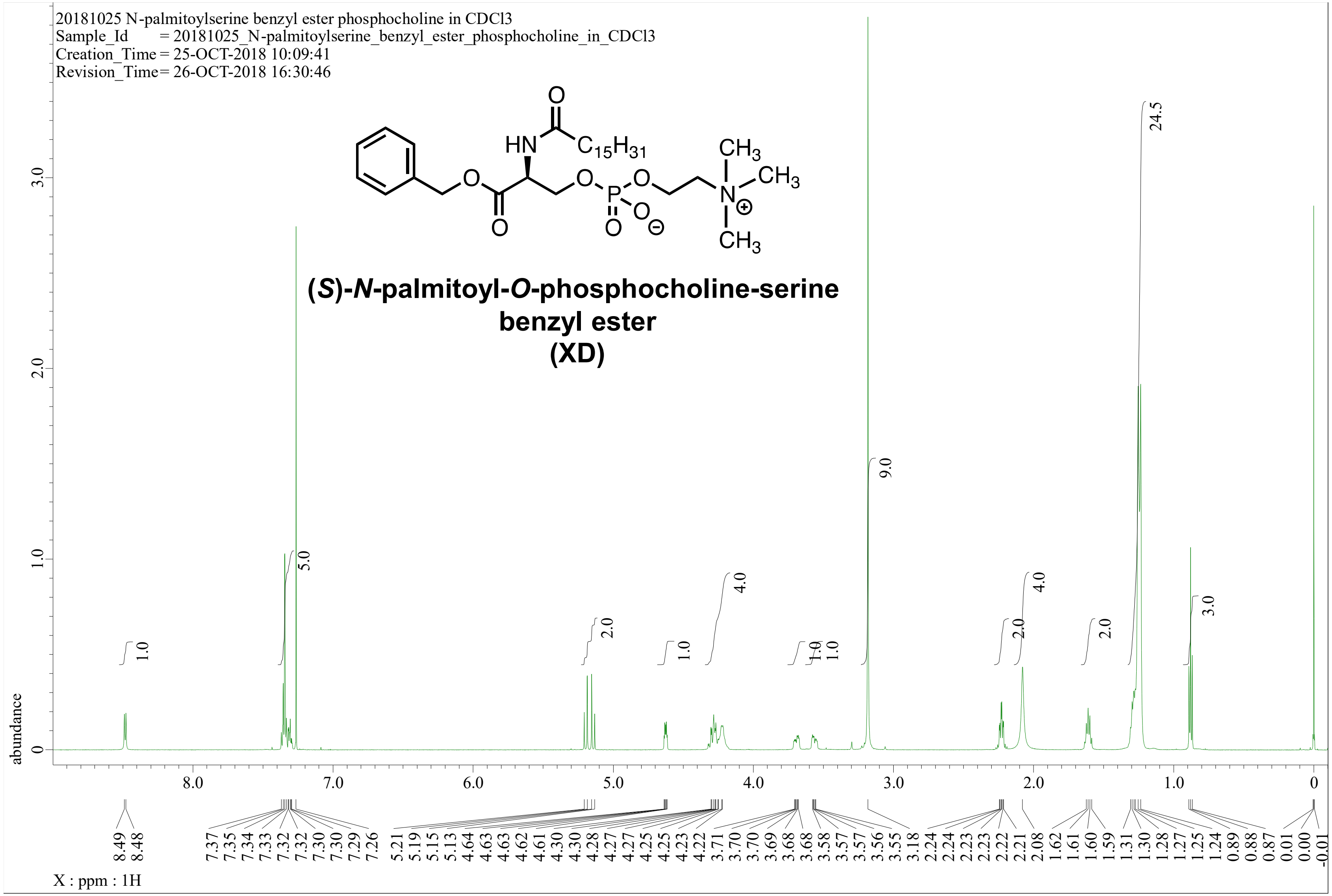

Fig. S3 (continued)

e, ^{1}H-NMR NMR spectrum of (*S*)-*N*-palmitoyl-*O*-phosphocholineserine benzyl ester (XD) in $CDCl_3$.

δ: 0.88 (t, 3H, J = 6.9 Hz, $-CH_3$), 1.24–1.31 (m, 24H, palmitoyl), 1.60 (m, 2H, NCO-CH_2-CH_2), 2.19–2.27 (m, 2H, NCO-CH_2), 3.18 (s, 9H, $-N(CH_3)_3$), 3.56 (qd, 1H, J = 6.6, 2.7 Hz, $-POCH_2CH_2N$), 3.69 (qd, 1H, J = 6.6, 2.7 Hz, $-POCH_2CH_2N$), 4.22–4.30 (m, 4H, $-CH_2OPOCH_2-$), 4.61–4.64 (m, 1H, -COCHN-), 5.17 (dd, J = 31.6, 13.1 Hz, 2H, Bn-CH_2-OCO), 7.29–7.37 (m, 5H), 8.48 (d, 1H, J = 6.9 Hz, NH);

F

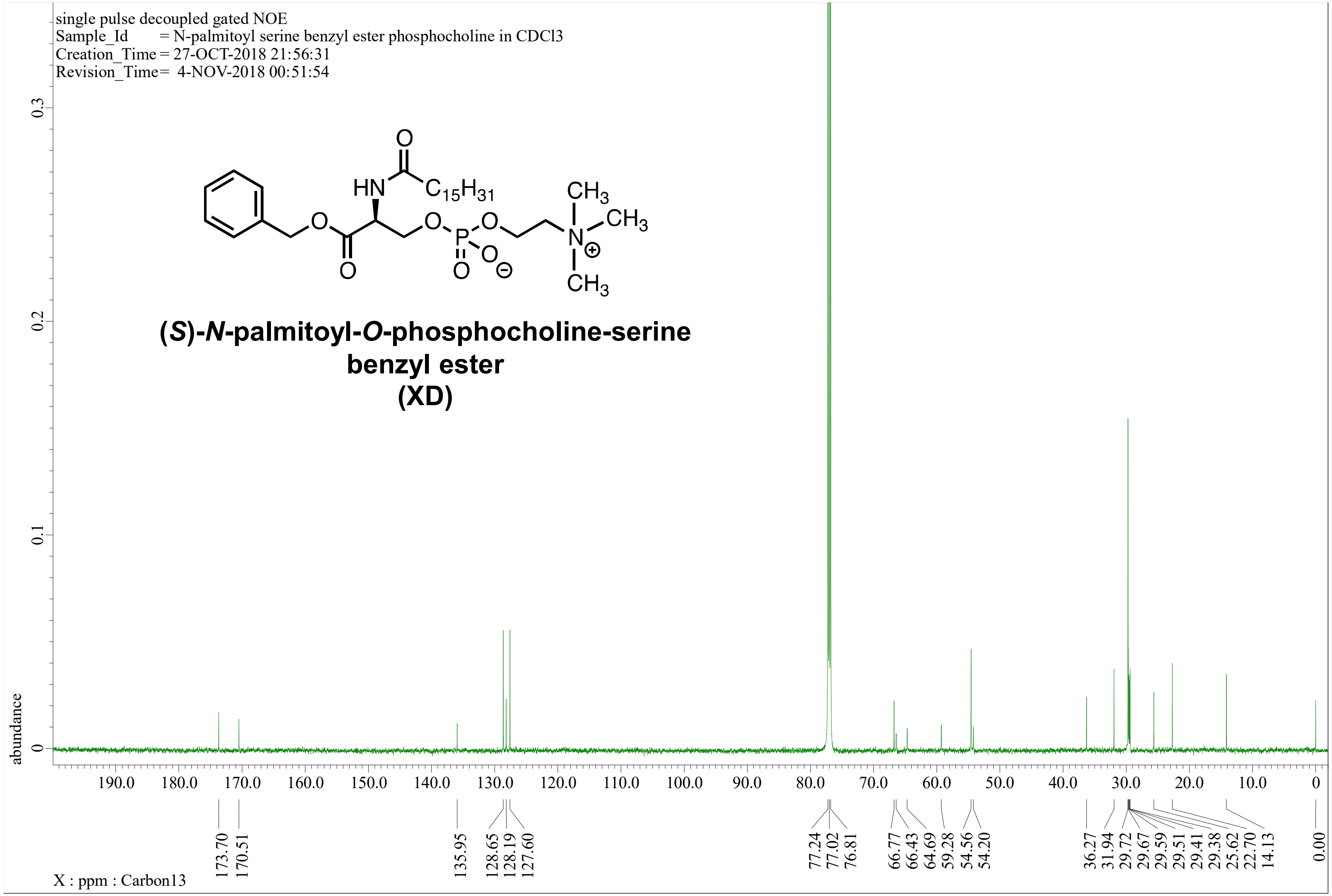

Fig. S3(continued)

f, ^{13}C-NMR NMR spectrum of (*S*)-*N*-palmitoyl-*O*-phosphocholineserine benzyl ester (XD) in $CDCl_3$.

δ: 14.13 (-CH_3), 22.70 (-CH_2CH_3), 25.62 (-$NHCOCH_2CH_2$-), 29.4–29.7 (multiple peaks in the range), 31.9 (-$CH_2CH_2CH_3$), 36.3 (-$NHCOCH_2$-), 54.2 (-NHCH-), 54.6 (-$N(CH_3)_3$), 59.3 (-$POCH_2CH_2$-), 64.7 (-$CHCH_2OP$-), 66.4 (-$POCH_2CH_2N$-), 66.8 ($BnCH_2$-), 127.6 (Ph), 128.2 (Ph), 128.7 (Ph), 136.0 (quaternary carbon in Ph), 170.5 (BnOCO-), 173.7 (-NHCO-).

G

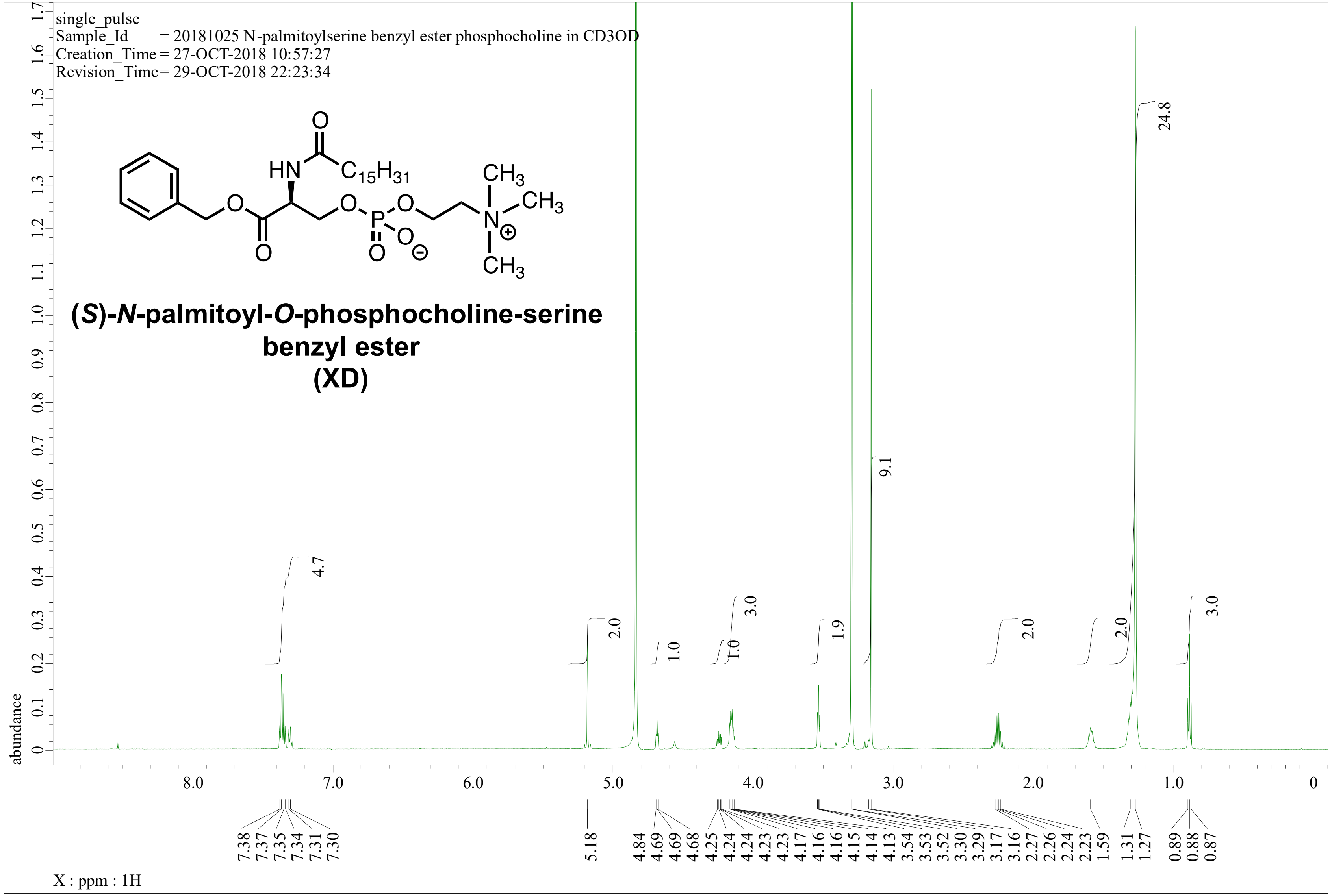

Fig. S3(continued)

g, ¹H-NMR NMR spectrum of (*S*)-*N*-palmitoyl-*O*-phosphocholineserine benzyl ester (XD) in CD_3OD.

δ : 0.88 (t, 3H, J = 6.9 Hz, $-CH_3$), 1.27–1.32 (m, 25H, palmitoyl), 1.55–1.62 (m, 2H, NCO-CH_2-CH_2), 2.25 (qd, J = 14.8, 7.7 Hz, 2H, NCO-CH_2), 3.16 (s, 9H, $-N(CH_3)_3$), 3.53 (t, J = 4.8 Hz, 2H, $-POCH_2CH_2N$), 4.13–4.18 (m, 3H, $-CH_2OPOCH_2CH_2N$-), 4.23-4.26 (m, 1H, $-POCH_2CH_2N$-), 4.69 (t, J = 4.1 Hz, 1H, -COCHN-), 5.18 (t, J = 13.1 Hz, 2H, Bn-CH_2-OCO), 7.29–7.38 (m, 5H);

H

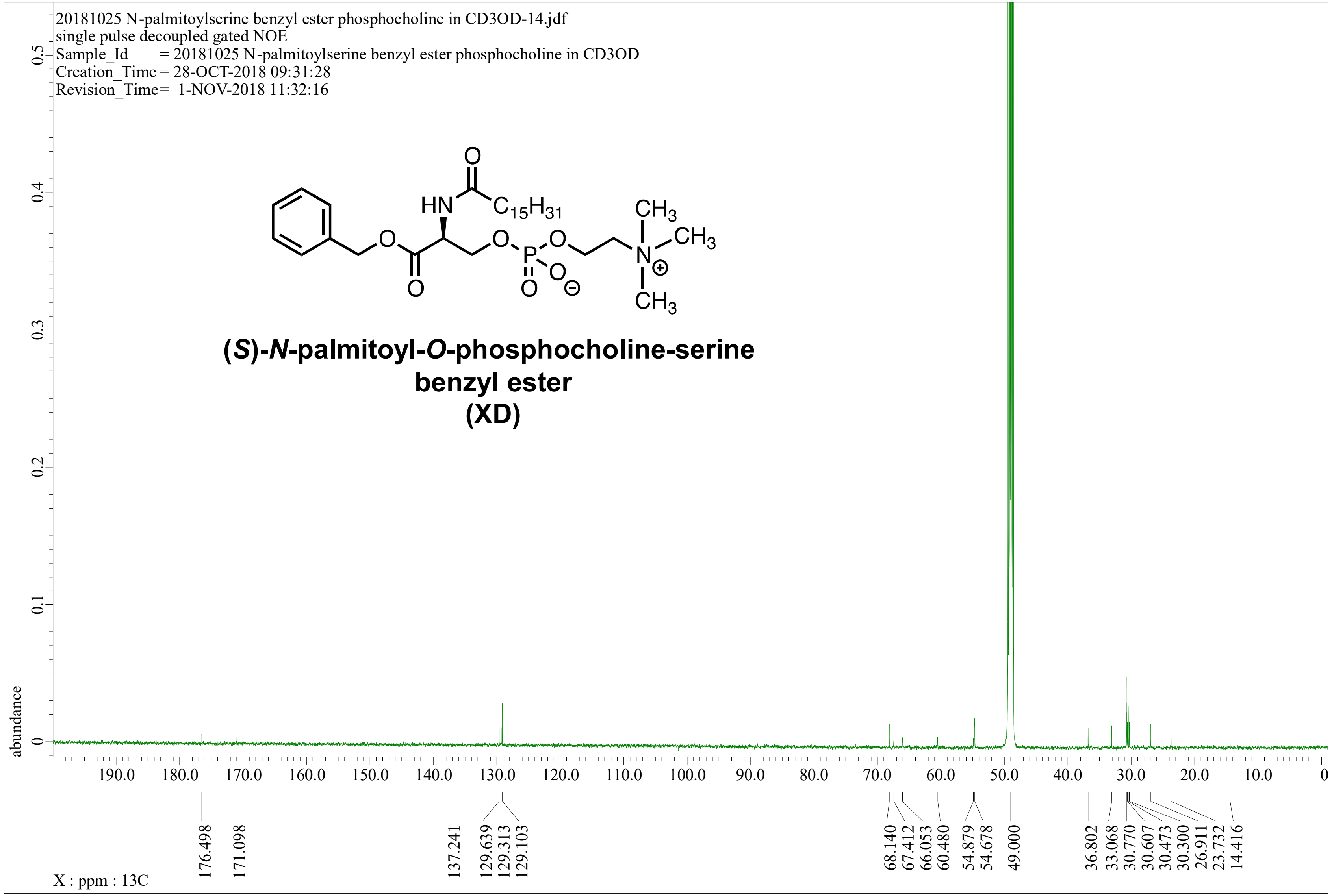

Fig. S3(continued)

h, ^{13}C-NMR spectrum of (*S*)-*N*-palmitoyl-*O*-phosphocholineserine benzyl ester (XD) in CD_3OD.

δ: 14.4 (-CH_3), 23.7 (-CH_2CH_3), 26.9 (-$NHCOCH_2CH_2$-), 30.3–30.8 (multiple peaks in the range), 33.07 (-$CH_2CH_2CH_3$), 36.8 (-$NHCOCH_2$-), 54.7 (-NHCH-), 54.9 (-$N(CH_3)_3$), 60.48 (-$POCH_2CH_2$-), 66.1(-$CHCH_2OP$-), 67.4 (-$POCH_2CH_2N$-), 68.1 ($BnCH_2$-), 129.1 (Ph), 129.3 (Ph), 129.6 (Ph), 137.2 (quaternary carbon in Ph), 171.1 (BnOCO-), 176.5 (-NHCO-).

I

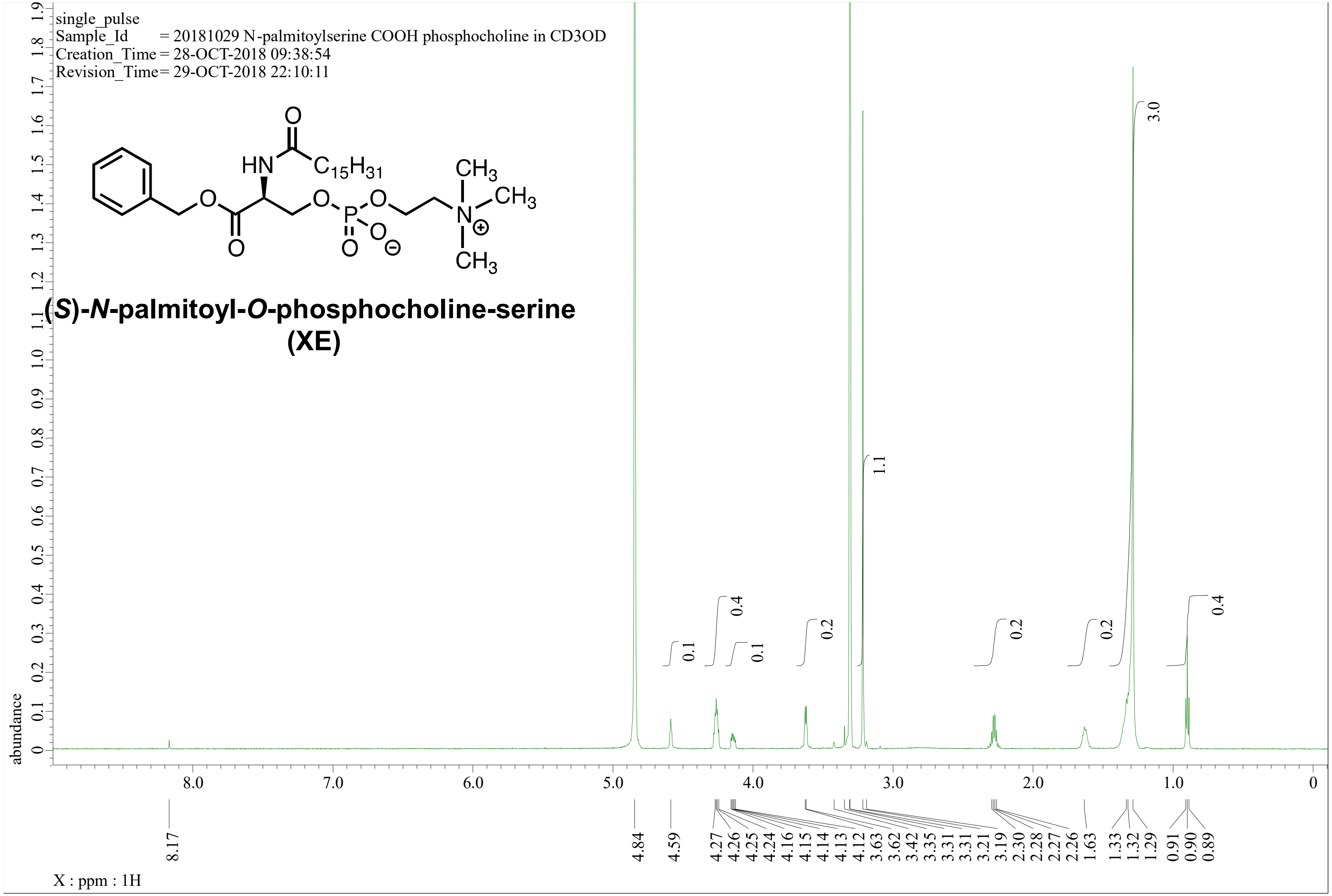

Fig. S3 (continued)

i, ^{1}H-NMR spectrum of (*S*)-*N*-palmitoyl-*O*-phosphocholine-serine in CD_3OD.

δ : 0.87 (t, J = 6.9 Hz, 3H, -CH_2CH_3), 1.26–1.31 (m, 24H, palmitoyl), 1.61 (s, 2H, NCO-CH_2-CH_2), 2.21–2.28 (m, 2H, -NCO-CH_2), 3.19 (s, 9H, -$N(CH_3)_3$), 3.60 (d, J = 4.8 Hz, 2H, -$POCH_2CH_2N$), 4.10–4.13 (m, 1H, -$CHCH_2OP$-), 4.22–4.25 (m, 3H, -CH_2OP(=O,-OH)OCH_2), 4.56 (s, 1H, -COCHN-).

J

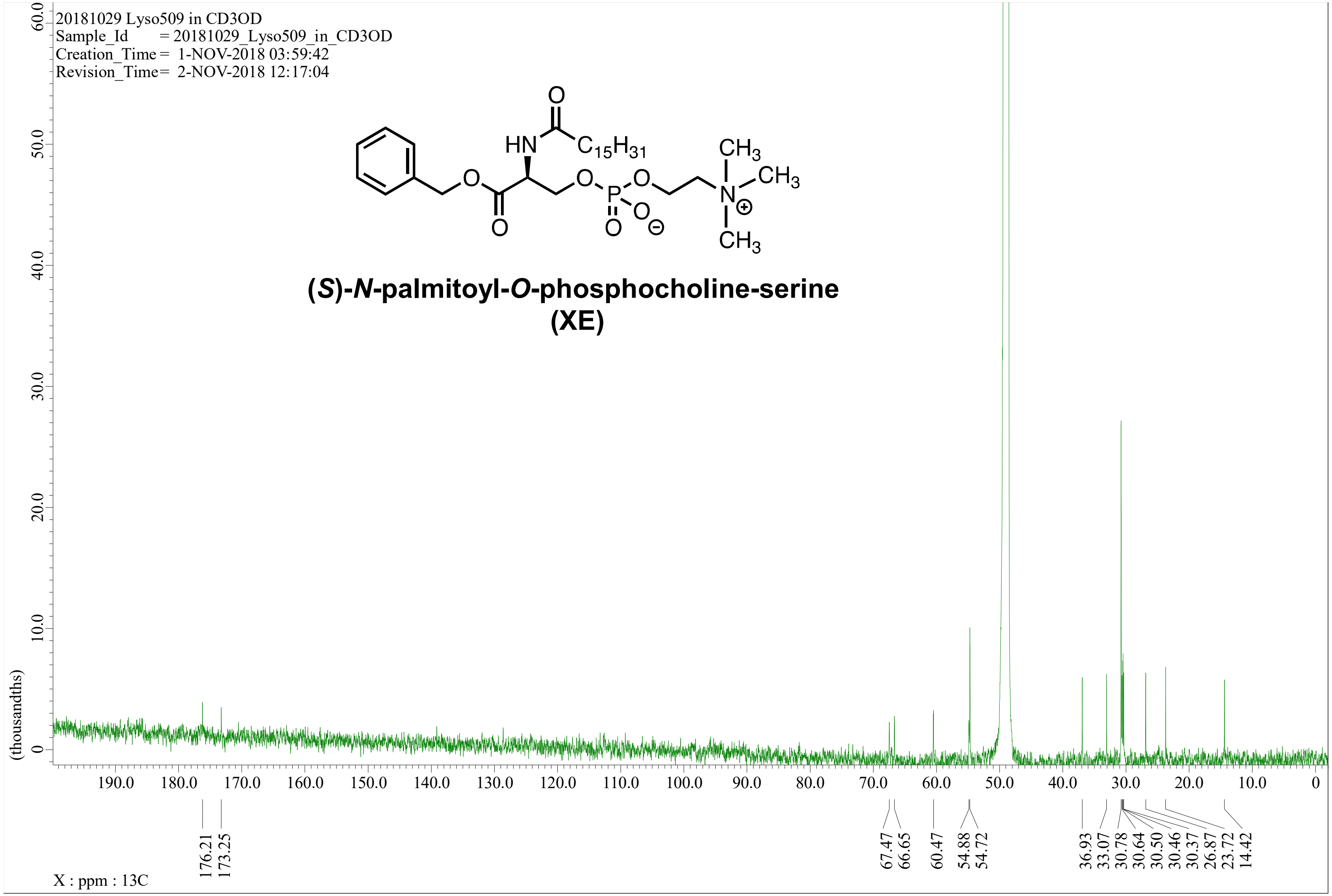

Fig. S3(continued).

j, ^{13}C-NMR spectrum of (*S*)-*N*-palmitoyl-*O*-phosphocholine-serine in CD_3OD.

δ : 14.4 (-CH_3), 23.7 (-CH_2CH_3), 26.87 (-$NHCOCH_2CH_2$-), 30.4–30.8 (multiple peaks in the range), 33.1(-$CH_2CH_2CH_3$), 36.9 (-$NHCOCH_2$-), 54.7 (-NHCH-), 54.9 (-$N(CH_3)_2$-), 60.5 (-$POCH_2CH_2$-), 66.7 (-$CHCH_2OP$-), 67.5 (-$CHCH_2OP$-), 173.3 (-COOH), 176.2 (-NHCO-).

K

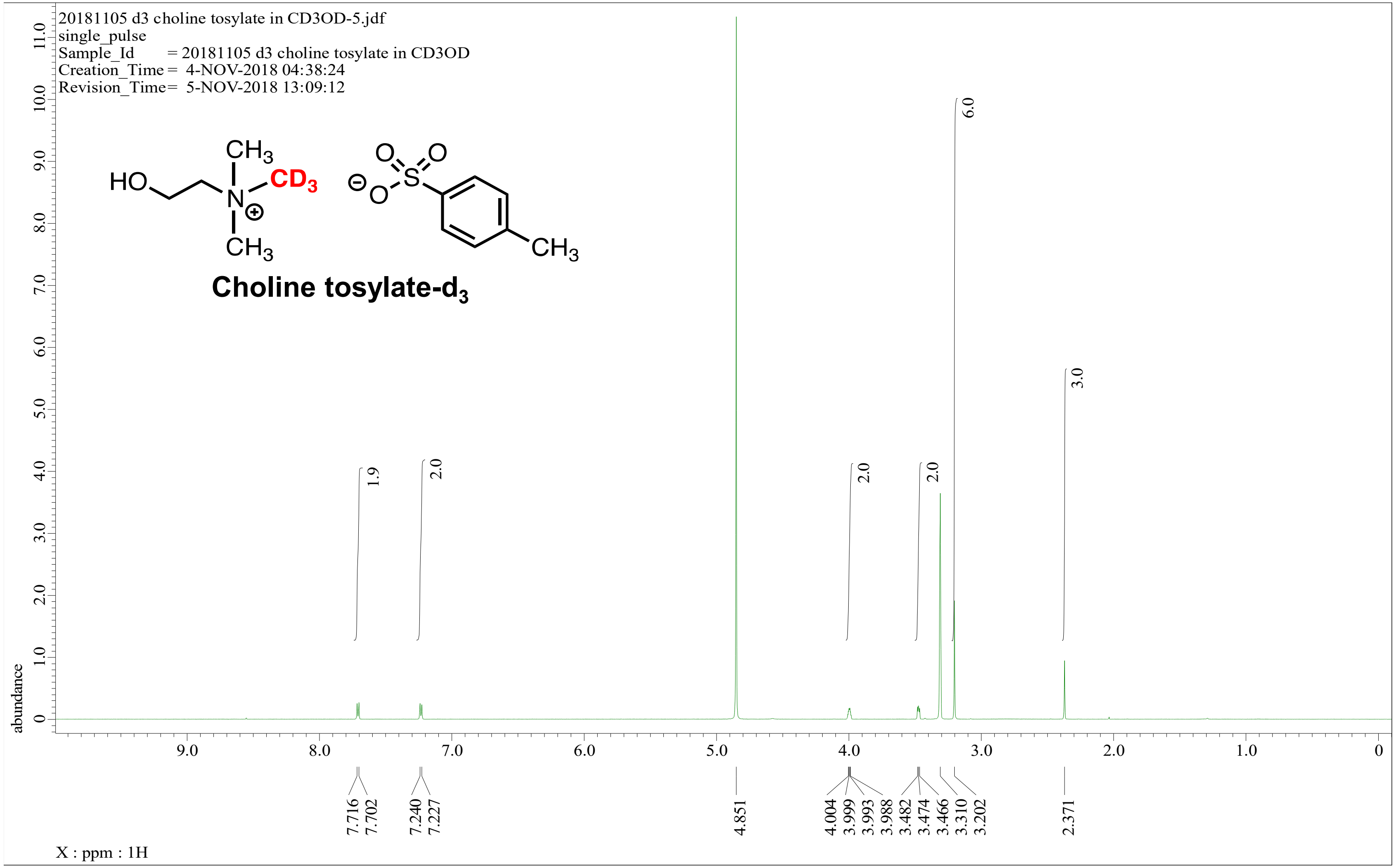

Fig. S3(continued).

k, ^{1}H-NMR of Choline tosylate-d_3 in CD_3OD.

δ 2.37 (s, 3H, Ar-CH_3), 3.20 (s, 6H, $N(CH_3)_2CD_3$), 3.47–3.48 (m, 2H), 3.98–4.01 (m, 2H), 7.23 (d, J = 8.2 Hz, 2H, Ar), 7.71 (d, J = 8.2 Hz, 2H, Ar).

L

M. Maekawa, I. Jinnoh, Y. Matsumoto, et al., Fig. S3

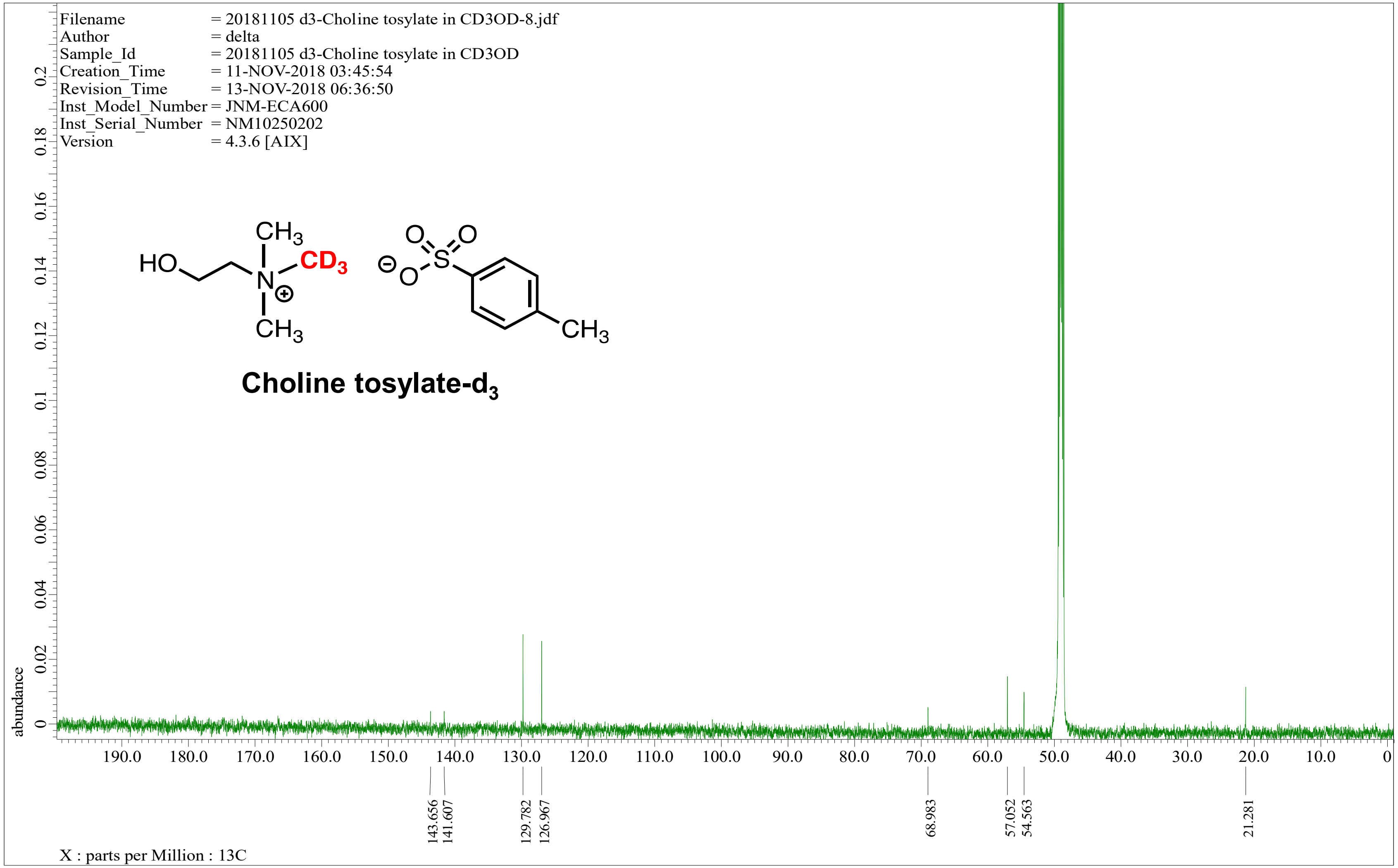

Fig. S3(continued).

I, ^{13}C-NMR of Choline tosylate-d_3 in CD_3OD

δ 21.3 (Ar-CH_3), 54.6 ($N(CH_3)_2CD_3$), 57.1 (NCH_2CH_2OH), 69.0 (NCH_2CH_2OH), 127.0 (CH_3-C=*C*H-), 129.8 (CH3-C-CH=*C*H), 141.6 (CH3-C), 143.7 (C-S).

M

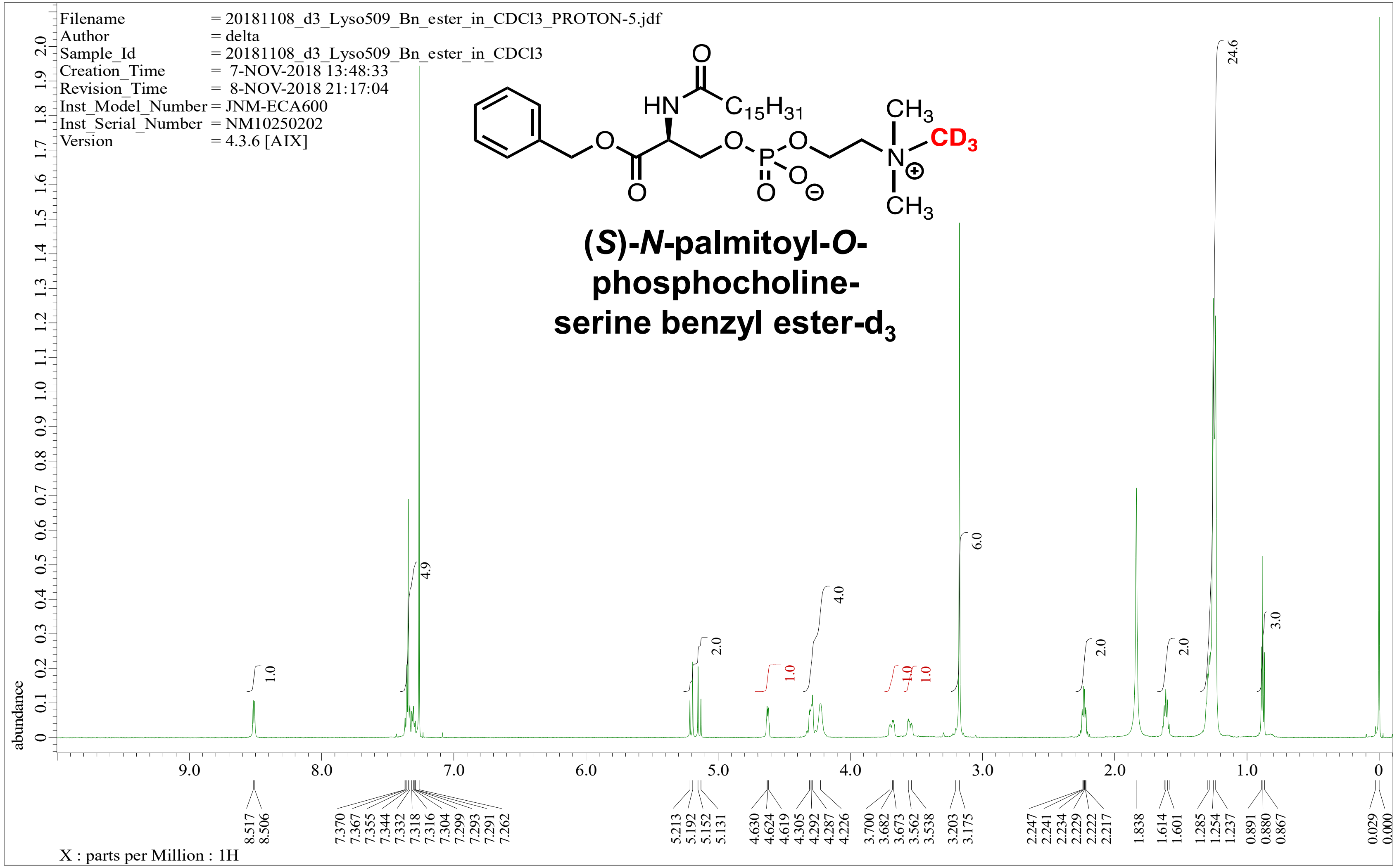

Fig. S3(continued).

m, ¹H-NMR of (*S*)-*N*-palmitoyl-*O*-phosphocholineserine benzyl ester-d$_3$ in $CDCl_3$

δ: 0.88 (t, J = 6.9 Hz, 3H, -CH_3), 1.24–1.30 (m, 25H, palmitoyl), 1.59–1.64 (m, 2H, -$NCOCH_2CH_2$-), 2.19–2.27 (m, 2H, NCO-CH_2), 3.18 (s, 6H, -$N(CH_3)_2$-), 3.53–3.58 (m, 1H, -$POCH_2CH_2N$), 3.67–3.71 (m, 1H, -POCH2CH2N), 4.23–4.31 (m, 4H, -CH_2OPOCH_2-), 4.61–4.64 (m, 1H, -COCHN-), 5.17 (dd, J = 36.8, 12.7 Hz, 2H, Bn-CH_2-OCO), 7.29–7.37 (m, 5H, Bn), 8.51 (d, J = 6.9 Hz, 1H, NH);

N

M. Maekawa, I. Jinnoh, Y. Matsumoto, et al., Fig. S3

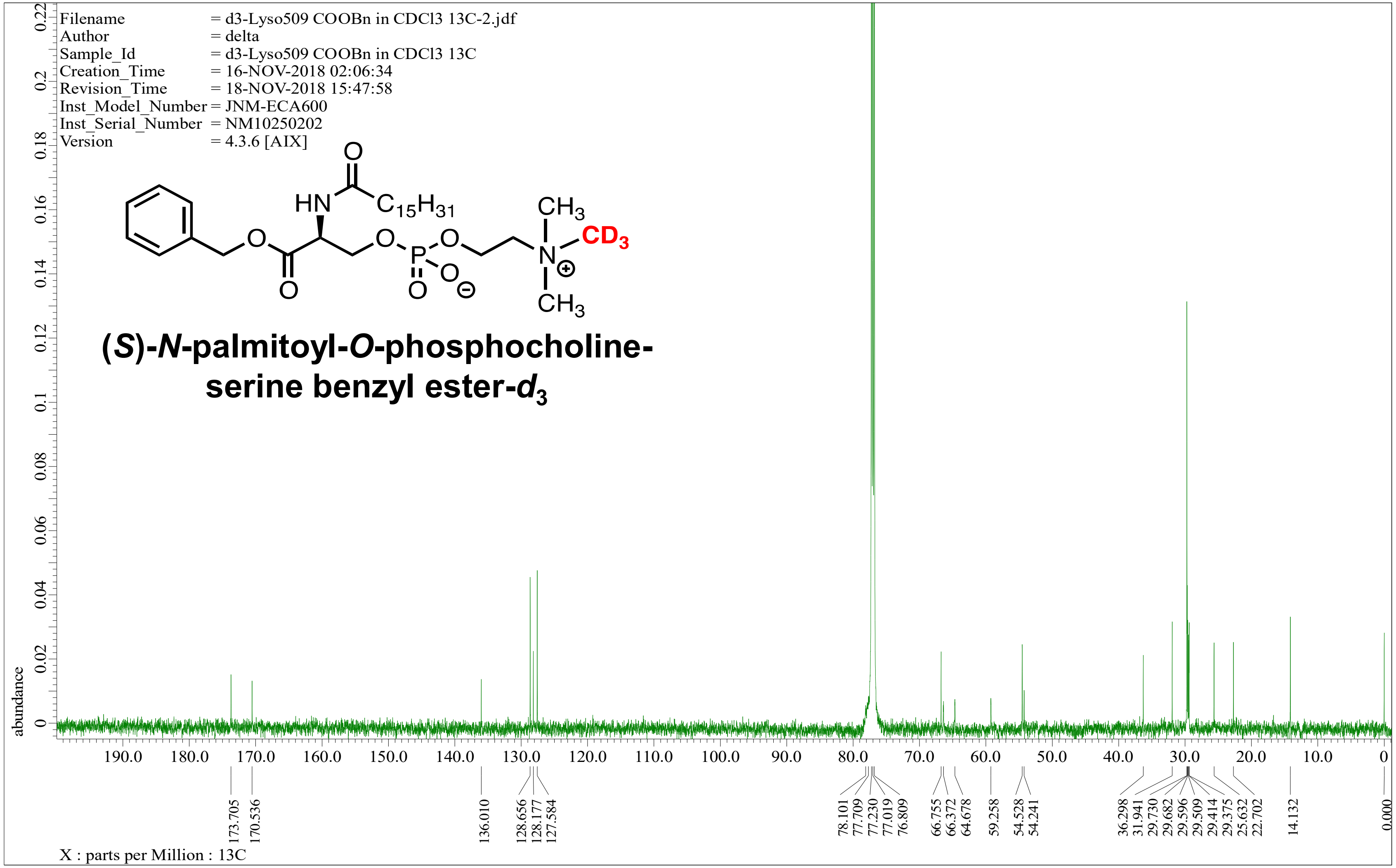

Fig. S3(continued).

n, ^{13}C- NMR of (*S*)-*N*-palmitoyl-*O*-phosphocholine-serine benzyl ester-d_3 in $CDCl_3$

δ: 14.13 (-CH_3), 22.70 (-CH_2CH_3), 25.6 (-$NHCOCH_2CH_2$-), 29.4–29.7 (multiple peaks in the range), 31.9 (-$CH_2CH_2CH_3$), 36.3 (-$NHCOCH_2$-), 54.2 (-NHCH-), 54.6 (-$N(CH_3)_3$), 59.3 (-$POCH_2CH_2$-), 64.7 (-$CHCH_2OP$-), 66.4 (-$POCH_2CH_2N$-), 66.8 ($BnCH_2$-), 127.6 (Ph), 128.2 (Ph), 128.7 (Ph), 136.0 (quaternary carbon in Ph), 170.5 (BnOCO-), 173.7 (-NHCO-).

o

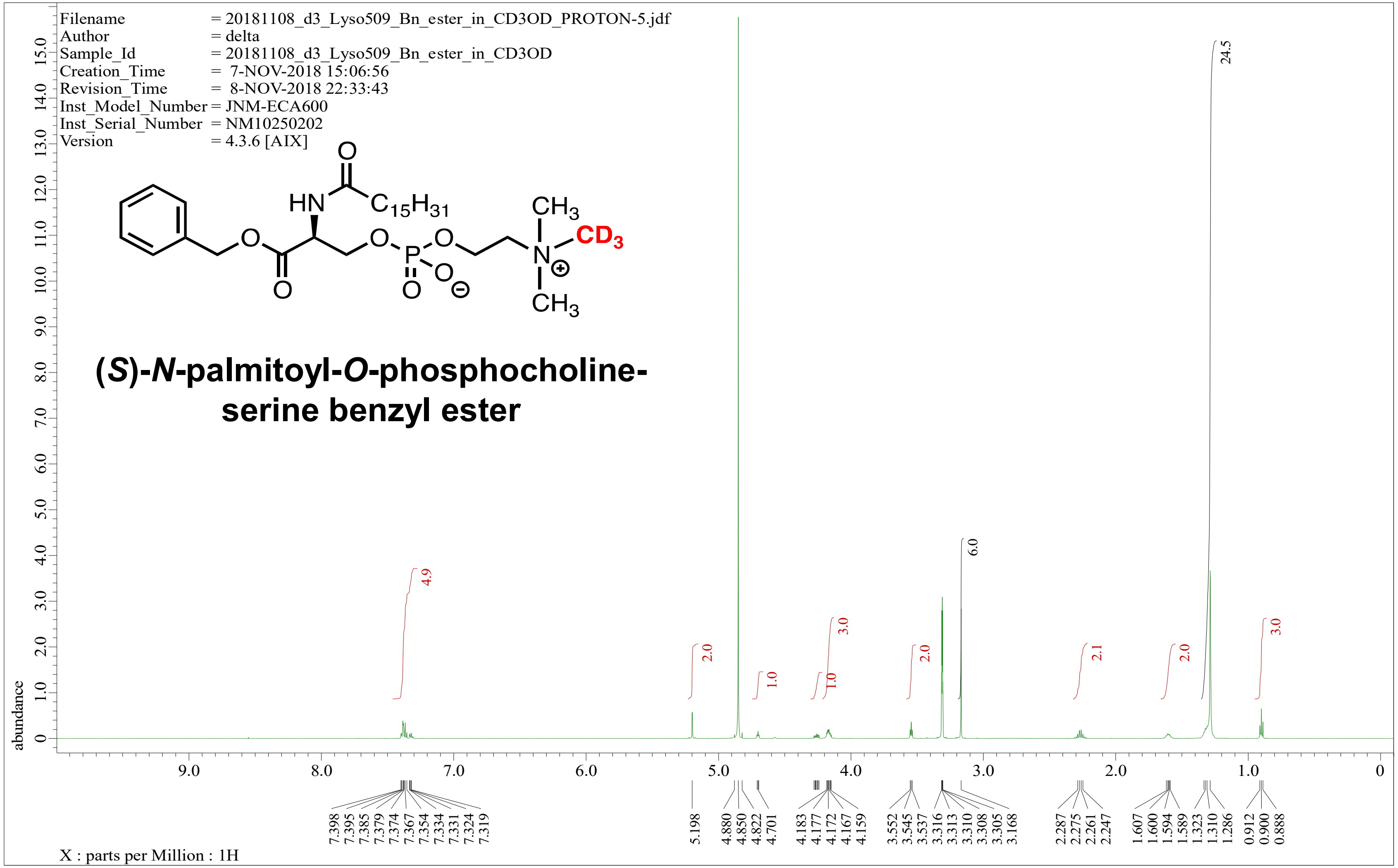

Fig. S3(continued).

o, ^{1}H-NMR of (*S*)-*N*-palmitoyl-*O*-phosphocholineserine benzyl ester in CD_3OD.

δ: 0.90 (t, J = 6.9 Hz, 3H, CH_3), 1.29-1.34 (m. 24H, palmitoyl), 1.58–1.64 (m, 2H, NCO-CH_2-CH_2), 2.27 (dq, J = 30 Hz, 7.2 Hz, 2H, -NCO-CH_2), 3.17 (s, 6H, -N$(CH_3)_2CD_3$), 3.54 (t, J = 4.8 Hz, 2H, -POCH2CH2N), 4.15–4.18(m, 3H, -CH2OP(=O,-OH)OCH_2) 4.24–4.28 (m, 1H, -CHCH2OP-), 4.58 (s, 0H, NH), 4.70 (t, J = 3.8 Hz, 1H, -COCHN-), 5.20 (t, J = 13.1 Hz,2H, Ar-CH_2), 7.31–7.40 (m, 5H, Ar).

P

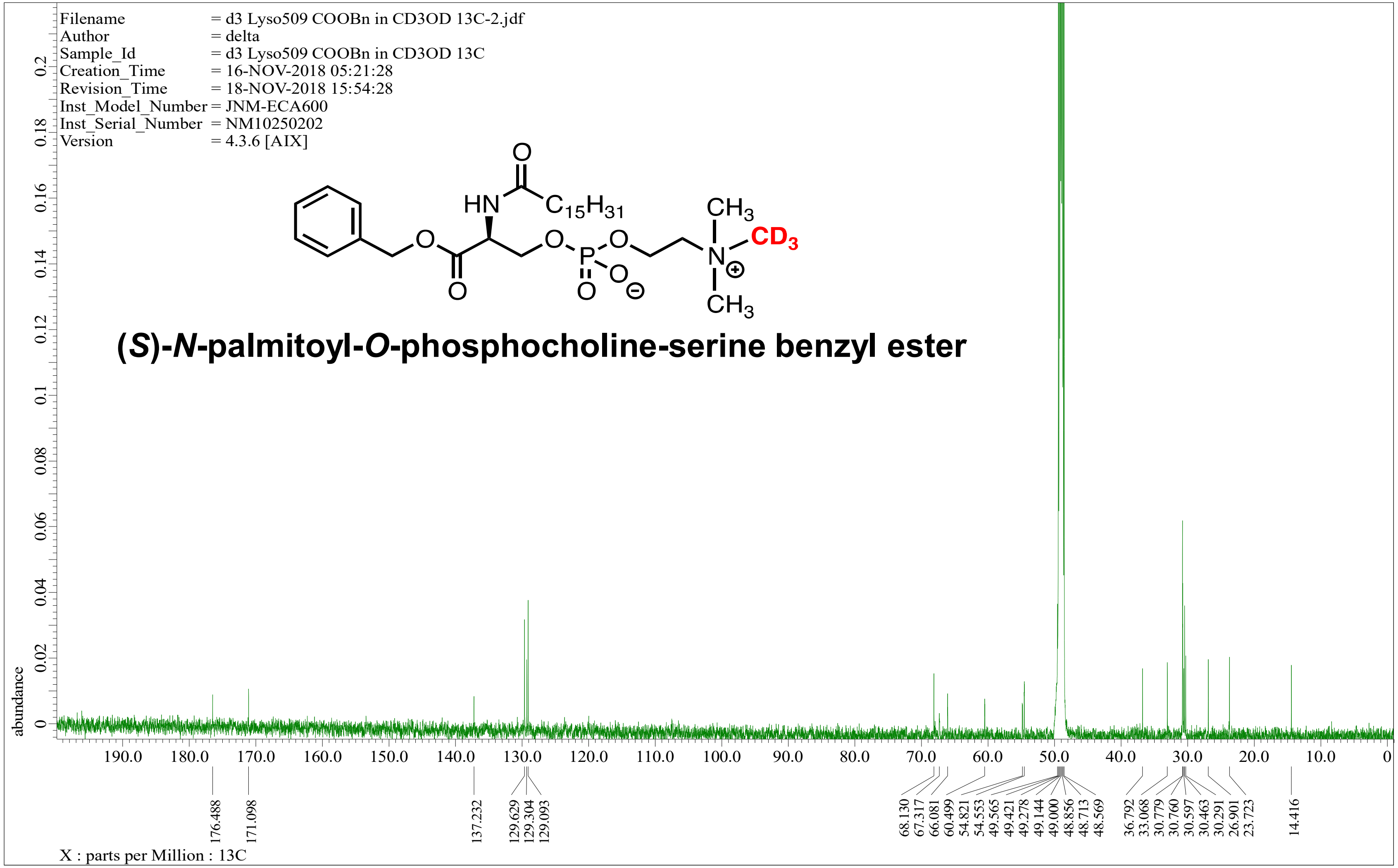

Fig. S3(continued).

p, ^{13}C-NMR of (*S*)-*N*-palmitoyl-*O*-phosphocholineserine benzyl ester in CD_3OD

δ: 14.1 (-CH_3), 22.7 (-CH_2CH_3), 25.6 (-$NHCOCH_2CH_2$-), 29.4-29.7 (multiple peaks in the range), 31.9 (-$CH_2CH_2CH_3$), 36.3 (-$NHCOCH_2$-), 54.2 (-$N(CH_3)_2$-), 54.5 (-NHCH-), 59.3 (-$POCH_2CH_2$-), 64.7 (-$CHCH_2OP$-), 66.4 (-$CHCH_2OP$-), 66.8 (-CHCH2OP-), 127.6 (Bn), 128.2 (Bn), 128.7 (Bn), 136.0 (Bn), 170.5 (COOH), 173.7 (NHCO).

Q

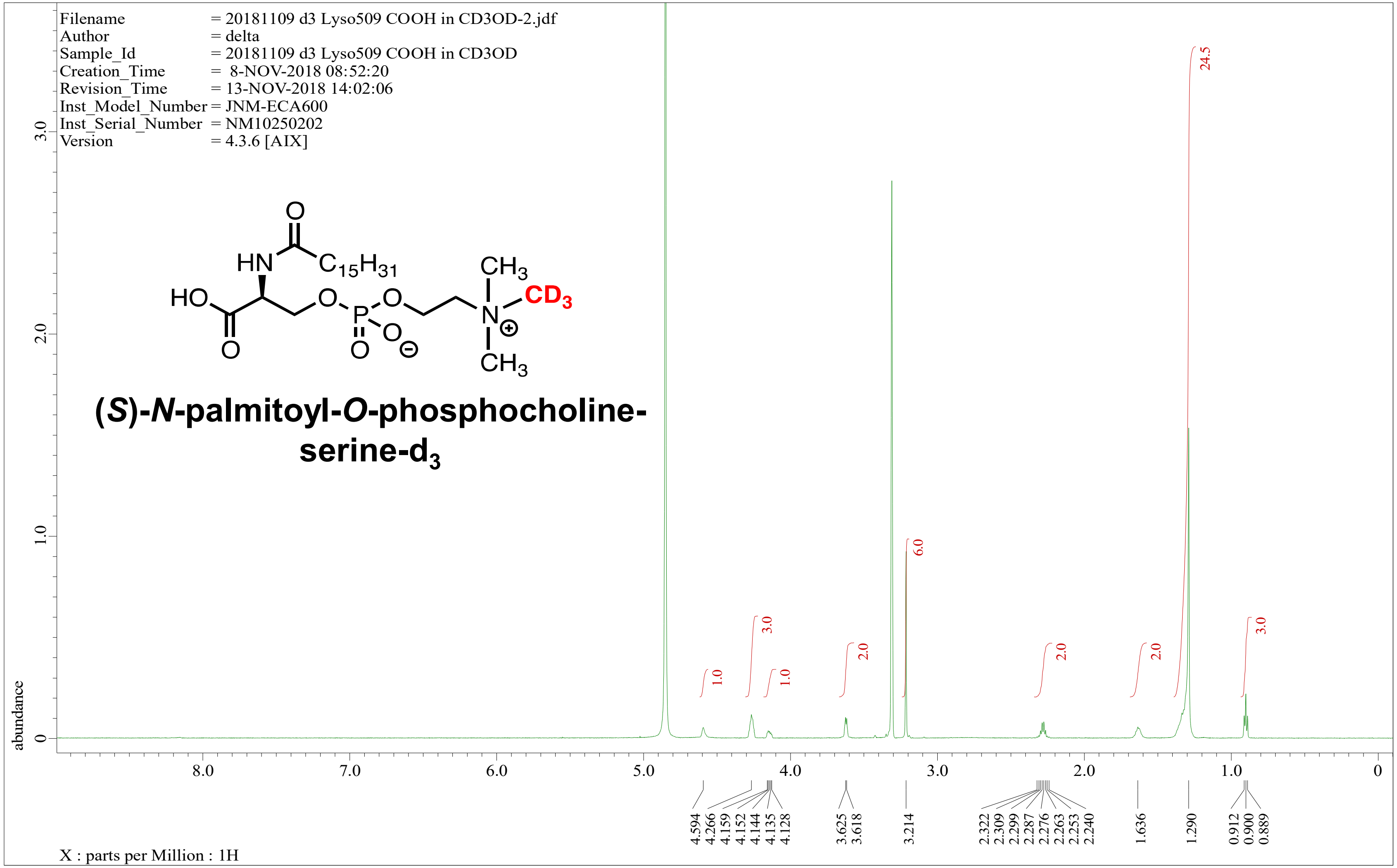

Fig. S3(continued).

q, ^{1}H-NMR of (*S*)-*N*-palmitoyl-*O*-phosphocholine-serine-d$_3$ in CD_3OD

δ: 0.90 (t, J = 6.9 Hz, 3H, CH_3), 1.29–1.34 (m, 24H, palmitoyl), 1.64 (m, 2H, NCO-CH2-CH2)), 2.24-2.30 (m, 2H, NCO-CH_2), 3.21 (s, 6H, -$N(CH_3)_2CD_3$), 3.62 (d, J = 4.1 Hz, 2H, -$POCH_2CH_2N$), 4.13–4.16 (m, 1H, -$CHCH_2OP$-), 4.26 (m, 3H, -CH_2OP(=O,-OH)OCH2), 4.59 (s, 1H, -COCHNH-);

R

M. Maekawa, I. Jinnoh, Y. Matsumoto, et al., Fig. S3

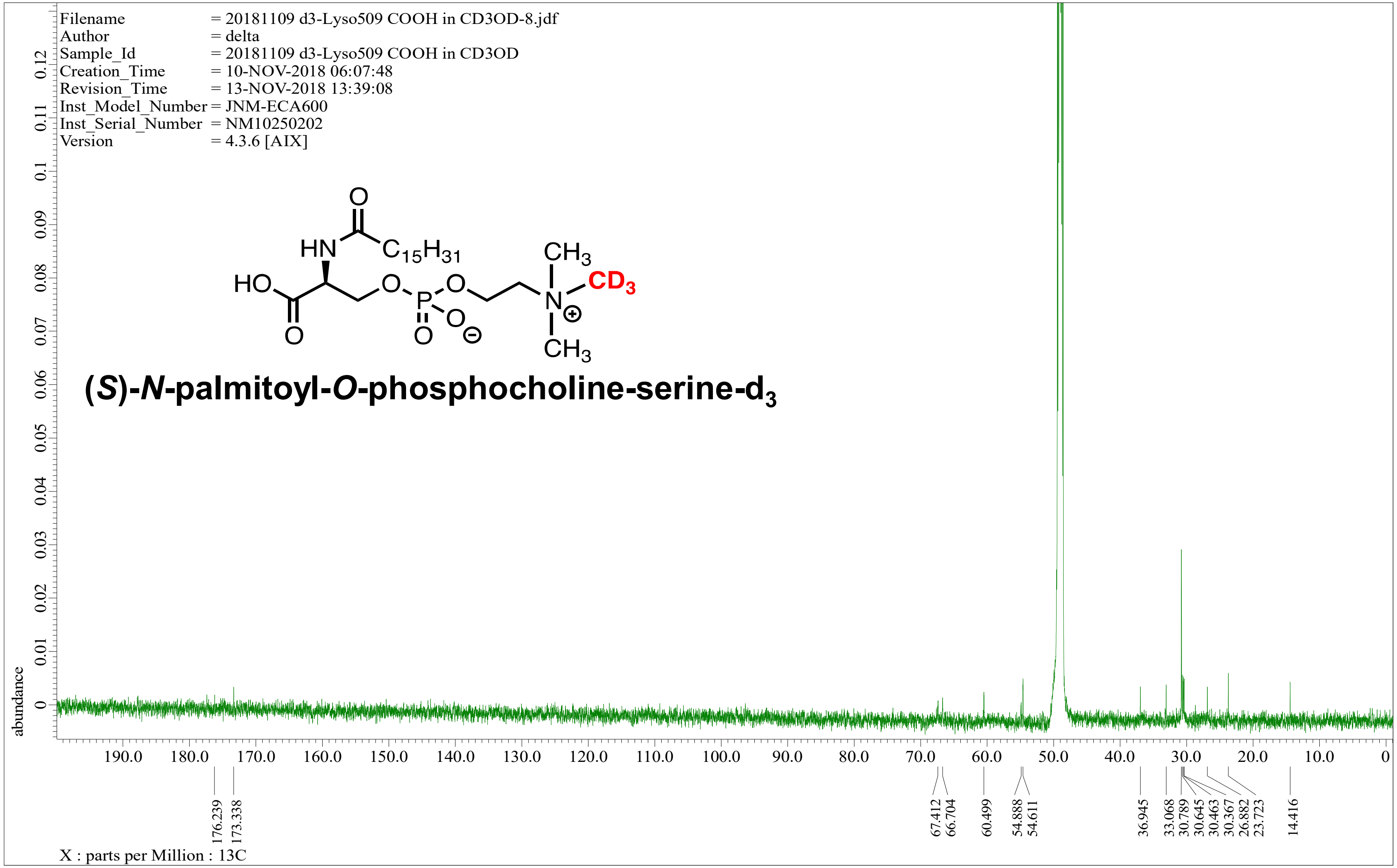

Fig. S3(continued).

r, ^{13}C- NMR of (*S*)-*N*-palmitoyl-*O*-phosphocholineserine (Lyso SM 509) in CD_3OD

δ: 14.4 (-CH_3), 23.7 (-CH_2CH_3), 26.9 (-$NHCOCH_2CH_2$-), 30.4–30.8 (multiple peaks in the range), 33.1 (-CH2CH2CH3), 36.9 (-$NHCOCH_2$-), 54.7 (-N(CH3)2CD3), 54.9 (-NHCH-), 60.5 (-$POCH_2CH_2$-), 66.7 (-$CHCH_2OP$-), 67.5 (-$CHCH_2OP$-), 173.3 (-COOH), 176.2 (-NHCO-).

Table S1 The serum/plasma concentrations of *N*-palmitoyl-*O*-phosphocholine-serine and SPC of patients with NPC and control subjects

Subject	Gender	Age (years)	Sample	*N*-Palmitoyl-*O*-phosphocholine-serine (ng/mL)	SPC (ng/mL)
NPC01	Female	24	Serum	2560	17.27
NPC02	Male	34	Serum	2010	8.25
NPC03	Female	52	Serum	2460	11.8
NPC04	Female	26	Serum	1080	8.11
NPC05	Female	21	Serum	1560	7.5
NPC06	Female	18	Serum	2410	12.57
NPC07	Female	11	Serum	3160	11.56
NPC08	Male	2.5	Serum	2510	8.11
NPC09	Male	0.8	Serum	1790	7.75
NPC10	Male	0.3	Serum	2270	17.51
NPC11	Female	11	Serum	3030	13.19
NPC12	Male	48	Serum	1920	12.66
NPC13	Female	30	Serum	1040	9.91
NPC14	Male	1.5	Serum	3410	17.2
NPC15	Male	28	Serum	9440	6.78
GAU01	Male	19	Serum	22.5	4.55
GAU02	Male	16	Serum	12.3	2.86
GAU03	Male	21	Serum	8.77	5.79
HUN01	Male	18	Serum	35.9	4.29
HUN02	Male	8	Serum	14.4	2.06
POP01	Female	23	Serum	15.7	3.29
NPS01	Female	26	Serum	7.35	3.67
NPS02	Female	0.0833	Serum	38.1	1.8
NPS03	Female	51	Serum	7.99	3.88
NPS04	Male	11	Serum	33.7	1.89
NPS05	Male	1.5	Serum	7.87	2.55
NPS06	Male	37	Serum	34.6	2.47
NPS07	Female	52	Serum	644	6.87
NPS08	Female	45	Serum	9.77	3.64
NPS09	Male	42	Serum	9.89	5.54

NPS10	Female	33	Serum	22.7	3.66
NPS11	Female	46	Serum	11.6	2.51
NPS12	Female	8	Serum	14.6	3.77
NPS13	Female	0.056	Serum	17.2	2.46
NPS14	Female	23	Serum	12.7	4.07
NPS15	Female	24	Serum	10.3	4.11
NPS16	Female	7	Serum	17.7	4.53
NPS17	Female	36	Serum	40.3	2.04
NPS18	Female	0.5	Serum	27.2	3.34
NPS19	Female	19	Serum	55.5	2.2
NPS20	Female	12	Serum	11.9	3.47
NPS21	Female	15	Serum	14.0	3.8
NPS22	Female	34	Serum	8.85	4.2
NPS23	Male	43	Serum	28.3	5.03
NPS24	Female	52	Serum	14.2	2.33
NPS25	Male	2	Serum	16.2	3.45
NPS26	Female	42	Serum	23.1	3.17
NPS27	Female	57	Serum	18.3	2.26
NPS28	Male	14	Serum	14.9	3.87
NPS29	Male	17	Serum	11.5	4.44
NPS30	Male	61	Serum	64.5	3.91
NPS31	Male	3	Serum	4.36	2.34
NPS32	Female	39	Serum	15.6	3.06
NPS33	Male	14	Serum	19.1	3.68
NPS34	Male	48	Serum	16.2	3.64
NPS35	Male	17	Serum	12.2	3.17
NPS36	Female	5	Serum	10.6	1.96
NPS37	Male	32	Serum	10.4	2.22
NPS38	Female	69	Serum	17.6	3.29
NPS39	Female	58	Serum	29.4	4.47
NPS40	Female	11	Serum	8.21	2.7
NPS41	Male	54	Serum	63.9	4.38
NPS42	Female	39	Serum	10.6	4.14
NPS43	Female	14	Serum	11.5	3.16
HC01	Male	21	Serum	11.3	3.86
HC02	Male	22	Serum	20.7	5.16

HC03	Male	23	Serum	10.4	3.93
HC04	Male	23	Plasma	6.37	3.18
HC05	Male	24	Plasma	7.96	2.37
HC06	Male	24	Plasma	11.0	1.76
HC07	Male	24	Plasma	9.80	2.27
HC08	Male	25	Plasma	11.3	2.48
HC09	Male	26	Plasma	10.6	2.28
HC10	Male	26	Plasma	6.54	2.09
HC11	Male	26	Plasma	25.0	3.59
HC12	Male	27	Plasma	21.2	2.69
HC13	Male	27	Plasma	14.6	2.53
HC14	Male	27	Plasma	16.6	2.01
HC15	Male	27	Plasma	11.6	2.32
HC16	Male	28	Plasma	12.4	2.1
HC17	Male	29	Serum	14.4	4.82
HC18	Male	30	Plasma	13.7	3.36
HC19	Male	30	Plasma	17.6	2.9
HC20	Male	30	Plasma	25.5	3.5

GAU, patient with Gaucher disease; HC, healthy control; HUN, patient with Hunter disease; NPC, patient with Niemann-Pick disease type C; NPS, patients suspected for NPC but were not determined the mutations of *NPC1* and *NPC2*. POP, patient with Pompe disease.

Table S2 MS/MS parameters in selected reaction monitoring analysis for simultaneous quantitation.

No	Compound	Target	Q1 (*m/z*)	Q3 (*m/z*)	DP (V)	EP (V)	CE (V)	CXP (V)
1	*N*-Palmitoyl-*O*-phosphocholine-serine	Analyte 1	509.3	184.0	91	12	31	18
2	*N*-Palmitoyl-*O*-phosphocholine-serine-d_3	IS 1	512.3	184.0	91	12	31	18
3	SPC	Analyte 2	465.3	184.0	86	2	35	12
4	Lyso-SM (d17:1)	IS 2	451.3	184.0	86	2	35	12

CE, collision energy; CXP, collision cell exit potential; DP, declustering potential; EP, entrance potential; SPC, sphingosylphosphorylcholine (also called as Lyso-SM (d18:1)); *nor*-SPC, *nor*-sphingosylphosphorylcholine (also called as Lyso-SM (d17:1)); IS, internal standard.

Table S3 Analytical method validation.

(A) Matrix factor.

		Matrix factor (%)		IS normalized matrix factor (%)	
Target	Compound	LQC	HQC	LQC	HQC
Analyte 1	*N*-Palmitoyl-*O*-phosphocholine-serine	94.6	97.5	90.0	99.9
IS 1	*N*-Palmitoyl-*O*-phosphocholine-serine-d_3	105	97.6		
Analyte 2	SPC	102.5	99.1	98.2	100.1
IS 2	Lyso-SM (d17:1)	104.3	99.0		

Matrix factor (%) = $\frac{(Peak\ area\ of\ spiked\ serum)-(peak\ area\ of\ blank\ serum)}{(Peak\ area\ of\ standard\ solution)} \times 100$

IS normalized matrix factor (%) = $\frac{(Matrix\ factor\ of\ each\ analytes)}{(Matrix\ factor\ of\ IS)} \times 100$

Analyte 1 was normalized with IS 1 and Analyte 2 was normalized with IS 2.

(B) Calibration curve.

Compound	Quantification range (ng/mL)	Regression equation	Correlation coefficient
N-Palmitoyl-*O*-phosphocholine-serine	1–4000	y=0.00108x+0.00446	0.9974
SPC	1–4000	y=0.00085x+0.000177	0.9922

(C) Intra-assay and inter-assay reproducibility in serum.

	Precision (%)				Accuracy (%)		
Intra-day assay	Blank	LQC	MQC	HQC	LQC	MQC	HQC
N-Palmitoyl-*O*-phosphocholine-serine	2.27	0.670	1.57	0.997	-3.45	-0.610	-7.54
SPC	3.07	1.79	2.02	2.62	-2.13	-0.3	1.3
Inter-day assay	Precision (%)				Accuracy (%)		
Compound	Blank	LQC	MQC	HQC	LQC	MQC	HQC
N-Palmitoyl-*O*-phosphocholine-serine	1.50	1.35	0.400	0.399	-4.17	-1.25	-6.49
SPC	2.06	0.516	0.189	0.937	-2.95	-0.923	1.98

Precision was evaluated as relative standard deviation (R.S.D.).

$$\text{R.S.D. (\%)} = \frac{(Standard\ deviation)}{(Mean\ concentration)} \times 100$$

Recovery was evaluated as relative error (R.E.).

$$\text{R.E. (\%)} = \frac{(Calculated\ concentration) - ((Added\ concentration) + (Blank\ concentration))}{(Added\ concentration) + (Blank\ concentration)} \times 100$$

LQC, low quality control (2 ng/mL); MQC, middle quality control (80 ng/mL); HQC, high quality control (3000 ng/mL) .

(D) Stability test in serum.

	Recovery (%, Mean±SD)					
	Freeze and thaw		-80°C for 1 week		4°C for 24 h	
	LQC	HQC	LQC	HQC	LQC	HQC
N-Palmitoyl-*O*-phosphocholine-serine	104±0.26	99.1±0.681	101±2.47	97.6±1.05	100±0.134	95.7±0.935
SPC	102±2.80	97.1±1.97	98.2±1.66	96.9±2.43	99.4±1.45	97.1±2.64
	24°C for 12 h		Autosampler for 48 h			
	LQC	HQC	LQC	HQC		
N-Palmitoyl-*O*-phosphocholine-serine	105±2.08	97.1±4.39	105±1.56	92.9±0.442		
SPC	103±1.48	96.3±4.05	107±2.08	101±1.02		

LQC, low quality control (2 ng/mL); HQC, high quality control (3000 ng/mL).

(E) Intra-assay and inter-assay reproducibility in plasma.

	Precision (%)				Accuracy (%)		
Intra-day assay	Blank	LQC	MQC	HQC	LQC	MQC	HQC
N-Palmitoyl-*O*-phosphocholine-serine	2.27	0.670	1.57	0.997	-3.45	-0.610	-7.54
SPC	3.07	1.79	0.917	1.92	-2.13	11.7	17.5
Inter-day assay	Precision (%)				Accuracy (%)		
Compound	Blank	LQC	MQC	HQC	LQC	MQC	HQC
N-Palmitoyl-*O*-phosphocholine-serine	1.50	1.35	0.400	0.399	-4.17	-1.25	-6.49
SPC	2.06	0.516	0.0741	0.399	-2.95	12.4	18.9

Precision was evaluated as relative standard deviation (R.S.D.).

$$\text{R.S.D. (\%)} = \frac{(Standard\ deviation)}{(Mean\ concentration)} \times 100$$

Recovery was evaluated as relative error (R.E.).

$$\text{R.E. (\%)} = \frac{(Calculated\ concentration) - ((Added\ concentration) + (Blank\ concentration))}{(Added\ concentration) + (Blank\ concentration)} \times 100$$

LQC, low quality control (2 ng/mL); MQC, middle quality control (80 ng/mL); HQC, high quality control (3000 ng/mL).

(F) Stability test in plasma.

	Recovery (%, Mean±SD)					
	Freeze and thaw		-80°C for 1 week		4°C for 24 h	
	LQC	HQC	LQC	HQC	LQC	HQC
N-Palmitoyl-*O*-phosphocholine-serine	110±1.36	101±1.63	110±4.76	101±1.28	108±2.63	96.3±4.55
SPC	101±1.63	101±0.833	100±2.95	102±0.453	103±3.86	102±2.49
	24°C for 12 h		Autosampler for 48 h			
	LQC	HQC	LQC	HQC		
N-Palmitoyl-*O*-phosphocholine-serine	109±2.26	99.0±2.03	112±3.77	93.9±3.26		
SPC	96.7±1.89	100±1.81	101±1.83	101±3.05		

LQC, low quality control (2 ng/mL); HQC, high quality control (3000 ng/mL).

Table S4 MS/MS conditions for targeted lipidomics analysis.

Compound	Q1 (*m/z*)	Retention time (min)	IS
Lyso-PC (14:0)	468.3	22.2	NAOPCS (16:0)-d_3
Lyso-PC (16:1)	494.3	23.5	NAOPCS (16:0)-d_3
Lyso-PC (16:0)	496.3	26.2	NAOPCS (16:0)-d_3
Lyso-PC (18:1)	522.4	27.2	NAOPCS (16:0)-d_3
Lyso-PC (18:0)	524.4	29.7	NAOPCS (16:0)-d_3
Lyso-PC (20:1)	550.4	30.4	NAOPCS (16:0)-d_3
Lyso-PC (20:0)	552.4	33.0	NAOPCS (16:0)-d_3
SPC	465.3	19.2	*nor*-SPC
NAOPCS (14:0)	481.3	19.3	NAOPCS (16:0)-d_3
NAOPCS (16:0)	509.3	23.5	NAOPCS (16:0)-d_3
NAOPCS (18:0)	537.4	27.19	NAOPCS (16:0)-d_3
NAOPCS (20:0)	565.4	30.62	NAOPCS (16:0)-d_3
nor-SPC	451.3	16.95	NAOPCS (16:0)-d_3
NAOPCS (16:0)-d_3	512.3	23.53	NAOPCS (16:0)-d_3

Lyso-PC, lysophosphatidylcholine; NAOPCS, *N*-acyl-*O*-phosphocholine-serine, SPC, sphingosylphosphorylcholine (also called as lyso-sphingomyelin (d18:1)); *nor*-SPC, *nor*-sphingosylphosphorylcholine (also called as lyso-sphingomyelin (d17:1))
